# TRIM21, a New Component of the TRAIL-Induced Endogenous Necrosome Complex

**DOI:** 10.3389/fmolb.2021.645134

**Published:** 2021-04-15

**Authors:** Mélanie Simoes Eugénio, Florence Faurez, Ghania H. Kara-Ali, Mélanie Lagarrigue, Perrine Uhart, Marion C. Bonnet, Isabelle Gallais, Emmanuelle Com, Charles Pineau, Michel Samson, Jacques Le Seyec, Marie-Thérèse Dimanche-Boitrel

**Affiliations:** ^1^ Univ-Rennes1, Inserm, EHESP, Irset (Institut de recherche en santé, environnement et travail) - UMR_S1085, Rennes, France; ^2^ Protim, Inserm, Irset - UMR_S1085, Campus de Beaulieu, Rennes, France; ^3^ Biogenouest, Core Facility Network in Western, France

**Keywords:** TRIM21, necroptosis, TRAIL, necrosome, proteomics

## Abstract

Tumor necrosis factor-related apoptosis-inducing ligand (TRAIL) is a well-known apoptosis inducer and a potential anticancer agent. When caspases and inhibitors of apoptosis proteins (IAPs) are inhibited, TRAIL induces necroptosis. Molecular mechanisms of necroptosis rely on kinase activation, and on the formation of a necrosome complex, bringing together the receptor-interacting protein kinases 1 and 3 (RIPK1, RIPK3), and the mixed lineage kinase domain-like protein (MLKL). In this study, mass spectrometry approach allowed to identify the tripartite motif containing 21 (TRIM21), an E3 ubiquitin-protein ligase as a new partner of the endogenous TRAIL-induced necrosome. Alteration of TRIM21 expression level, obtained by transient transfection of HT29 or HaCat cells with TRIM21-targeted siRNAs or cDNA plasmids coding for TRIM21 demonstrated that TRIM21 is a positive regulator of TRAIL-induced necroptosis. Furthermore, the invalidation of TRIM21 expression in HT29 cells by CRISPR-Cas9 technology also decreased cell sensitivity to TRAIL-induced necroptosis, a shortcoming associated with a reduction in MLKL phosphorylation, the necroptosis executioner. Thus, TRIM21 emerged as a new partner of the TRAIL-induced necrosome that positively regulates the necroptosis process.

## Introduction

The tumor necrosis factor (TNF)-related apoptosis-inducing ligand (TRAIL) belongs to the TNF superfamily ligands. TRAIL binds to death receptors DR4 and DR5, resulting in the formation of a death inducing signaling complex leading to caspase−8 or −10 activation and apoptosis (Zamaraev et al., 2014). Apart from apoptosis induction, TRAIL can trigger necroptosis, a caspase-independent cell death program (Holler et al., 2000; Jouan-Lanhouet et al., 2012). In a comparable way to TNF-induced necroptosis signaling (Vanden Berghe et al., 2014), necroptosis initiated by TRAIL is characterized by the formation of a stable cytosolic complex termed necrosome, which includes at least receptor-interacting serine/threonine-protein kinases 1 and 3 (RIPK1 and RIPK3), mixed lineage kinase domain-like pseudokinase (MLKL), Fas-associated with death domain protein (FADD) and caspase-8.

TRAIL emerged as an interesting tool for cancer therapy since it selectively induces apoptosis in cancer cells, while sparing normal cells (Ashkenazi et al., 1999; Walczak et al., 1999). However, an important number of cancer cells remains resistant to TRAIL-induced death, due to high expression of anti-apoptotic factors (Newsom-Davis et al., 2009), such as inhibitors of apoptosis proteins (IAPs) (Vasilikos et al., 2017).

Several second mitochondrial-derived activator of caspases (SMAC) mimetics (SMs) have been developed to sensitize resistant cancer cells to apoptosis by inhibiting IAPs. SMs alone sensitize cancer cells to TRAIL-induced apoptosis (Fulda et al., 2002) whereas a combination of TRAIL with SMs and z-VAD-fmk, a pan-caspase inhibitor, activates necroptosis (He et al., 2009). In this study, the combination of TRAIL/z-VAD-fmk/Birinapant (TzB) was used to better characterize the necroptotic death signal induced by TRAIL. Affinity purification combined with mass spectrometry (AP-MS) analysis of the TRAIL-induced endogenous RIPK3-containing necrosome protein complex showed the presence of the known interactors RIPK3, RIPK1, MLKL, FADD, and caspase-8, but highlighted a new partner, TRIM21 (Tripartite Motif Containing 21), an E3-ubiquitin ligase. Furthermore, by using RNA interference or CRISPR-Cas9 technology, TRIM21 expression was, respectively, decreased or abolished, and conferred some resistance to TRAIL-induced necroptosis. On the contrary, overexpression of TRIM21 led to a sensitization of cells to TRAIL-induced death. Altogether, these data highlighted TRIM21 as a new partner and positive modulator of the TRAIL-induced necrosome.

## Materials and Methods

### Cell Culture

HT29 and HaCat cell lines were obtained from ATCC. HT29 cell lines were cultured in MEM (Gibco) supplemented with 10% Fetal Bovine Serum (FBS) (Eurobio), 100 U/ml penicillin and 100 µg/ml streptomycin (Gibco), 2 mM L-glutamine (Invitrogen) and MEM Non Essential Amino Acids 1X (Gibco). HaCat cell lines were cultured in Dulbecco’s Modified Eagle Medium (DMEM) (Gibco), supplemented with 10% of FBS (Hyclone), 100 U/ml penicillin and 100 µg/ml streptomycin and 2 mML-glutamine.

### Reagents and Antibodies

Recombinant human Superkiller TRAIL (TRAIL-SK), Necrostatin-1 (Nec1) and z-VAD-fmk were purchased from Enzo Life Sciences. Hoechst, propidium iodide (PI), dithiothreitol (DTT), iodoacetamide, ammonium bicarbonate and formic acid were obtained from Sigma-Aldrich. Protease inhibitor and PhosStop Phosphatase inhibitor cocktails were purchased from Roche. Dynabeads protein G, TNF, and SytoxGreen (SG) were obtained from Invitrogen (Life Technologies). Birinapant was from Selleck Chemicals and Necrosulfonamide (NSA) was from Calbiochem. The multi-aggregated Ig-CD95L (or Ig-FasL) was a gift from Dr. Patrick Legembre (CLCC Eugène Marquis, Rennes).

Used antibodies were antibodies to caspase-8 clone C15 (ALX-804-429) (Alexis Biochemicals), FADD (556402) (Becton Dickinson), TRIM21 E-11 (sc-48430), Hsc70 (sc-7298) (Santa-Cruz), β-actin (A5316) (Sigma), RIPK3 (48-909), DR4 (1167), and DR5 (2019) (ProSci Incorporated), RIPK1 (D94C12) XP (3493S) (Cell Signaling), phospho-MLKL (ab187091) (Abcam), RIPK3 (PA1-41533) (Pierce), phospho-RIP1 (Ser166) (D1L3S), and phospho-RIP3 (Ser227) (D6W2T) (Cell Signaling) (this rabbit monoclonal antibody to P-RIPK3 recognizes two bands on western blot), FADD (06711) (Millipore), goat polyclonal antibodies to RIPK3 (clone N-14) (sc-47368), TRIM21 M-20 (sc-21367) (Santa-Cruz) and rat monoclonal antibodies to MLKL (MABC604) (Millipore). Horseradish-peroxydase-conjugated secondary antibodies were provided by Dako and Clean Blot IP detection kit provided by Life Technologies.

### Small Interfering RNA Transfection

Cells cultured in 96-well plates were transfected by reverse transfection with double-stranded ON TARGETplus SMARTpool siRNAs targeting mRNAs of hTRIM21 (EG:6737) (GE Healthcare). A non-specific targeting siRNA (siNT1, control siRNA, GE Healthcare) was used as a negative control for all experiments. Eighty nM of siRNA was transfected per well using DharmaFECT-4 transfection reagent (ThermoFisher Scientific).

### Plasmid Transfection With Amaxa

HT29 and HaCat cells were transfected using 4D-Nucleofector^TM^ or 3D-Nucleofector^TM^ (Lonza), respectively, as per the manufacturer’s instructions with control plasmid (pcDNA3.1+/N-GST-[Thrombin], GeneScript) or TRIM21 plasmid (pcDNA3.1+/N-GST-[Thrombin-hTRIM21], GeneScript). Briefly, 10 µg of plasmids were added to a suspension of 1,000,000 cells diluted in the Lonza SF transfection buffer and submitted to the FF137 transfection program (HT29 cells) or in the Lonza Nucleofector Solution and submitted to the U-020 transfection program (HaCat cells). Cells were then seeded in 9.6 cm^2^ well containing fresh supplemented medium as described in the cell culture section. Necroptosis was induced 48 h later by TzB treatment for 24 h and analyzed by the MTS assay.

### Calcium Phosphate Mediated Plasmid Transfection to Rescue TRIM21 Expression

TRIM21-KO HT29 cells were seeded at 2,000,000 cells per well in a 6-well plate, and allowed to plate for 4–6 h. Three µg of DNA (pEGFP-N1-Ro52, a gift from Dr. Mary Wahren-Herlenius, Division of Rheumatology, Department of Medicine, Karolinska Insitutet, Stockholm, Sweden) or pEGFP-N1 vector (Clontech Laboratories) diluted in 125 µl of 2M CaCl2 were added to 125 µl of HBS 2X (280 mM NaCl; 10 mM KCl; 1.5 mM Na_2_HPO_4_; 12 mM dextrose; 50 mM HEPES; pH between 7.05 and 7.12). After 16 h of incubation with the mixture, cells were washed for 1 min with PBS supplemented with 10% dimethyl sulfoxide (DMSO) and then incubated at 37°C with fresh supplemented medium. Necroptosis was induced 24 h later by TzB treatment in MEM containing 2 µg/ml of PI. Cell death was monitored by immunofluorescence live imaging as described below.

### CRISPR-Mediated TRIM21 Knockout in HT29 Cell Line

Two gRNA targeting human TRIM21 sequences (AGC​ACG​CCT​TGA​CAA/TGA​TGTGGG and TGG​CTA​GCT​GTC​GA/TTG​GGCCGG) were designed using the CRISPR Design Tool from the Zhang Laboratory and cloned into the pSpCas9(BB)-2A-GFP (PX458) plasmid (Addgene # 48138, a gift from Feng Zhang) by BbsI digestion and ligation. One million of HT29 cells were transfected with 5 µg of both plasmids by electroporation using the Amaxa 4D technology (Synnanovect platform, Biosit, Rennes) as previously described. GFP positive cells were sorted by flow cytometry 48 h after electroporation (Cell Sorter Aria, Becton Dickinson). A monoclonal cell line, which does not express TRIM21, was obtained after serial dilutions in a 96-well plate.

### Cell Viability

Cell survival was determined using CellTiter 96^®^ AQ_ueous_ one solution cell proliferation assay kit (MTS assay, Promega) as previously described (Jouan-Lanhouet et al., 2012).

### Detection of Necrotic Cells

Microscopic detection of apoptosis or necroptosis was carried out in both floating and adherent cells using Hoechst 33342 (1 µg/ml) and PI (1 µg/ml) staining dyes as previously described (Jouan-Lanhouet et al., 2012). Flow cytometry analysis (FL-2) (FACScalibur, Becton Dickinson) was also used to detect necroptotic cells using PI staining dye (0.5 µg/ml).

### Real-Time Analysis of Necroptosis by Immunofluorescence Live Imaging

WT or TRIM21-KO HT29 cells were seeded at 30,000 cells per well in a 96-well plate and allowed to adhere for 24 h. The following day, cells were treated with TRAIL-SK (100 ng/ml) in the presence of z-VAD-fmk (25 µM), Birinapant (1 µM) (TzB), and SYTOX™ Green reagent (5 µM) diluted in MEM. Cell death kinetic was monitored with the IncuCyte S3® live-cell imaging system. Fluorescent and phase contrast images were taken every hours until 8 h post-treatment. Data of total integrated fluorescent signal intensity were generated by the IncuCyte® S3 Software and expressed as relative fluorescence units (RFU).

### Immunoprecipitation and Immunoblotting

Cell pellets were collected and lysed using RIPA buffer (50 mM Tris-HCl pH 7.4; 1% Triton X-100; 25 mM HEPES; 150 mM NaCl; 0.2% SDS; 5 mM MgCl_2_; 1 mM Na_3_VO_4_) completed with protease inhibitors (Complete™ Protease Inhibitor Cocktail, Roche) and phosphatase inhibitors (PhosSTOP™, Roche). After lysis and sonication, samples were centrifuged at 13,000 rpm for 10 min, at 4°C. For RIPK3 immunoprecipitation, 50,000,000 of cells were stimulated with 500 ng/ml TRAIL-SK for the indicated times at 37°C. Anti-RIP3 antibody (Pierce, PA1-41533) or IgG control were incubated with lysates for 3 h at 4°C. Then 25 µl of Protein G Dynabeads was added and left to incubate overnight at 4°C. After immunoprecipitation, magnetic beads were washed three times with lysis buffer and one time with PBS. Immunoprecipitated proteins were then recovered using elution buffer (200 mM Glycine pH 2.5). The acidic pH of the elution was neutralized by adding 1 M Tris-HCl, pH 8.

After Bradford quantification, immunoprecipitated proteins or lysates were separated by SDS-PAGE and transferred onto nitrocellulose membrane. Membranes were then blocked with 4% Bovine Serum Albumine (BSA) in TBS 1X (20 mM Tris; 137 mM NaCl), 0.1% (v/v) Tween 20 (TBS-Tween) during 1 h and then incubated overnight at 4°C with indicated primary antibodies. Membranes were then washed twice with TBS-Tween and incubated for 1 h with peroxidase-conjugated secondary antibody. Protein-antibody complexes were revealed by enhanced chemoluminescence (Millipore) and imager analysis (ChemiDoc XRS+, BioRad). The Image Lab™ software (BioRad) was used for western blot visualization.

### Mass Spectrometry: Identification of Immunoprecipitated Proteins

This experiment was realized in duplicate. For each time point treatment with TzB, 0 and 3 h, eight RIPK3 immunoprecipitates were pooled (240 µL) and subjected to enzymatic digestion: proteins were reduced with 25 µL of 65 mM DTT (15 min at 37°C) then alkylated with 25 µL of 135 mM iodoacetamide (15 min at room temperature in the dark). The sample was completed with 170 µL of 50 mM ammonium bicarbonate (pH 8.5) and finally digested with 2 µg of modified trypsin (Promega) for 5 h at 37°C. The peptide mixture was then desalted and concentrated using a micro spin-column C18 from Harvard Apparatus according to manufacturer’s instructions. The resulting sample was completely dried, then solubilized with 17 µL of 0.1% acid formic and injected in a nanoflow high-performance liquid chromatography (HPLC) system (Dionex, LC Packings Ultimate 3,000) connected to a hybrid LTQ-OrbiTrap XL (Thermo Fisher Scientific) equipped with a nano-electrospray ionization (ESI) source (New Objective). Mobile A [99.9% MilliQ water and 0.1% formic acid (v:v)] and B [99.9% acetonitrile and 0.1% formic acid (v:v)] phases for HPLC were delivered by an Ultimate 3,000 nanoflow LC system (Dionex, LC Packings). The sample (10 µL) was loaded onto a trapping precolumn (5 mm × 300 μm i. d., 300 Å pore size, Pepmap C18, 5 μm) during 3 min with 2% buffer B at a flow rate of 25 μL/min. Reverse-phase separation was then performed at a flow rate of 0.250 μL/min using an analytical column (15 × 300 μm i. d., 300 Å pore size, Pepmap C18, 5 μm, Dionex, LC Packings) thermostated at 30°C. A gradient from 2 to 35% buffer B for the first 60 min, 35 to 60% buffer B from 60 to 85 min, and 60 to 90% buffer B from 85 to 105 min was used. Finally, the column was washed with 90% buffer B for 16 min, and with 2% buffer B for 19 min. The peptides were directly eluted from the column into the ESI source of the mass spectrometer. A voltage of 1.6 kV was applied to the HPLC buffer using the liquid junction provided by the ESI source and the ion transfer tube temperature was set to 200°C. The LTQ-Orbitrap XL instrument was operated in the data-dependent mode by automatically switching between full scan MS and consecutive MS/MS acquisitions. Full scan MS spectra were acquired in the OrbiTrap with a resolution of 60,000 at m/z 400 in the mass range 400–2,000; ion injection times were calculated to allow the accumulation of 10^6^ ions in the OrbiTrap for each spectrum. The ten most intense ions (with an intensity ≥2,000 counts and a charge state ≥2) of each full scan MS were sequentially isolated and fragmented in the linear ion trap by collision-induced dissociation (normalized collision energy at 35%, activation time of 30 m). Peaks selected for fragmentation were automatically subjected to dynamic exclusion for 60 s with a mass tolerance of ±10 ppm to avoid the selection of the same ion for fragmentation more than once. For OrbiTrap measurements, an external calibration was used before each injection series ensuring an overall error mass accuracy below 5 ppm for the detected ions. MS data were saved in RAW file format (Thermo Fisher Scientific) using XCalibur 2.0.7 with tune 2.4.

Proteome Discoverer 1.2 software (Thermo Fisher Scientific) supported by Mascot (Mascot server v2.2.07; http://www.matrixscience.com) database search engine was used for peptide and protein identification using its automatic decoy database search to calculate a false discovery rate (FDR). MS/MS spectra were compared to the UniProt Human Reference proteome set database (UniProt release 2015_09, September 29, 2015, 70075 sequences, 23,655,377 residues). Mass tolerance for MS and MS/MS was set at 10 ppm and 0.5 Da, respectively. The enzyme selectivity was set to full trypsin with one miscleavage allowed. Protein modifications were fixed: carbamidomethylation of cysteines and variable oxidation of methionine.

### Identification Validation and Spectral Count Label-free Quantification

Proline Studio 1.1 software was used for the validation and the spectral count comparison of the identified proteins in each samples [http://proline.profiproteomics.fr/] (Carapito et al., 2015). After importation of the mascot. dat files from each query, search results were validated with a peptide rank = 1 and a FDR of 1% on the e-value at the peptide spectrum match level. Proteins identified with exactly the same set of peptides or with a subset of the same peptides were grouped in a Protein Set. This Protein Set is then represented by a Typical Protein which is the best-identified protein (best score) or in case of same set proteins, the SwissProt one if possible. When proteins with shared peptides were identified with other peptides not belonging to the Protein Set, different Protein Sets were created, even if there are no specific peptides (i.e., if theses peptides were also shared by other Protein Sets). For the spectral count comparison, a parent dataset corresponding to the merge of the individual validated identification result was created. This parent dataset is used to define the shared and specific peptides and the Protein Set list to compare. For each protein, we chose to calculate weighted spectral counts, as suggested in Abacus (Fermin et al., 2011), where shared peptides are combined and weighted according to the associated Protein Sets. Briefly, for each shared peptide, we define which proportion of spectra is allocated to the different Protein Sets. These weights take into account the specific spectral counts of the different Protein Sets sharing the same peptide(s). To detect significant difference between samples, a beta-binomial test was performed on these weighed spectral counts and a *p*-value was calculated for each Protein Set using the R package BetaBinomial 1.2 implemented in Proline Studio (Pham et al., 2010).

### Statistical Analysis

Three independent experiments, each consisting of three replicates, were performed for each assay. Student's *t*-test was used to compare means between different treatment groups. Statistical tests were performed using Prism 5.01 (GraphPad Software, San Diego, CA).

## Results

### Treatment With TRAIL/z-VAD-fmk/Birinapant (TzB) Induces Necroptosis and RIPK1/RIPK3 Interaction in Human HT29 Colon Carcinoma Cells

**FIGURE 1:**
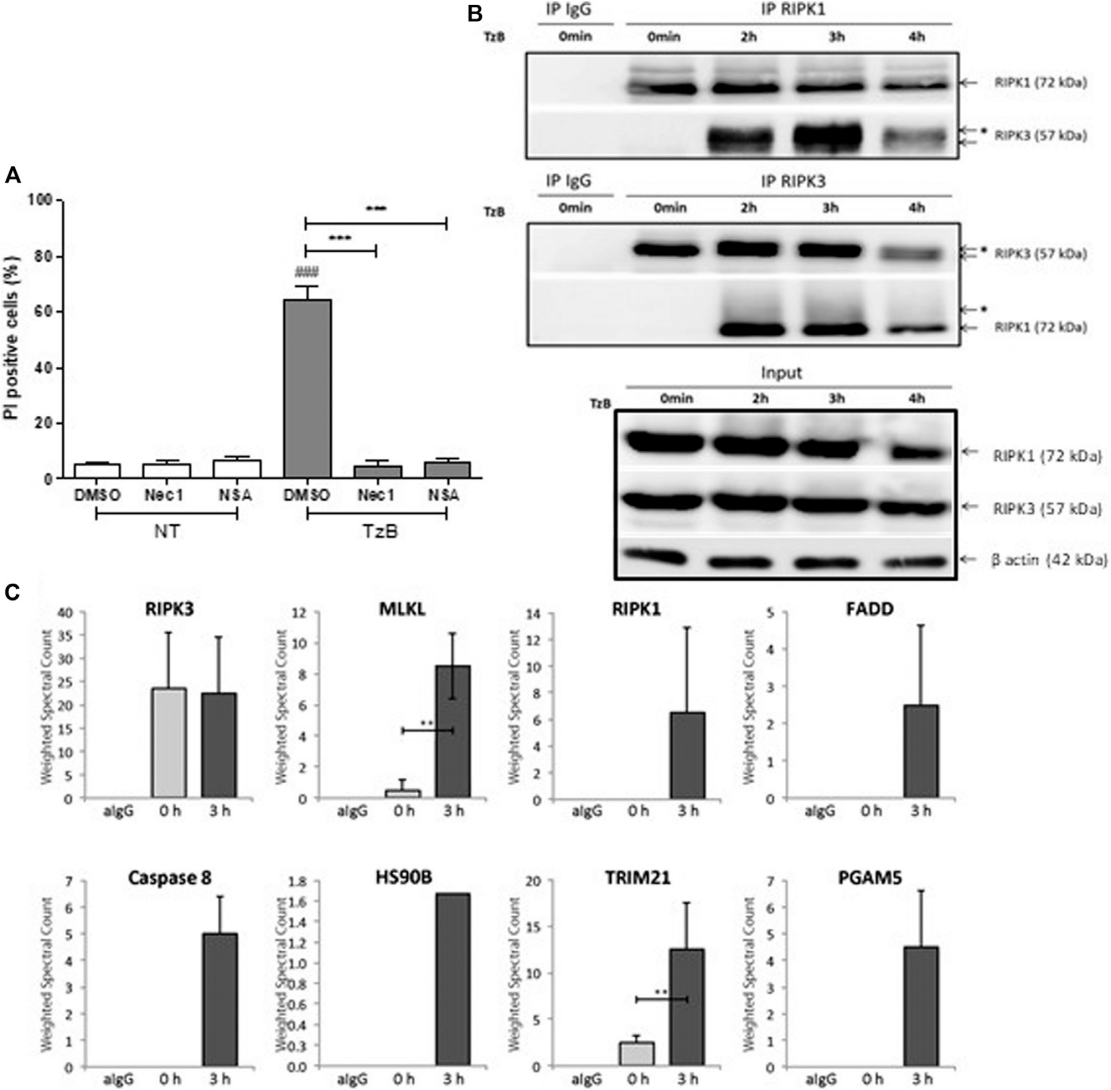
TRAIL/z-VAD-fmk/Birinapant (TzB) induces necroptosis and necrosome formation in HT29 cells. **(A)** HT29 cells were pretreated with vehicle (DMSO 0.1%), Necrostatin-1 (Nec1, 10 µM) or Necrosulfonamide (NSA, 1 µM) for 1 h. Cells were then treated with TzB (in grey) or not (NT, in white) for 24 h. Percentage of PI positive dead cells were measured by flow cytometry (mean ± SD, *n* = 3). (###), *p* < 0.001 compared treated cells to NT cells; (***), *p* < 0.001 compared treated conditions. **(B)** HT29 cells were treated or not (0 min) with TRAIL-SK (500 ng/ml), z-VAD-fmk (25 µM) and Birinapant (Bp, 1 µM) for the indicated times. Four mg of cell lysates were immunoprecipitated (IP) with RIPK1 or RIPK3 antibody or control IgG. The RIPK1 or RIPK3 immunocomplexes were analyzed by western blot. *: an upper band for RIPK3 or a smear for RIPK1. Anti-human β-actin antibody was used as protein loading control. Representative data of three independent experiments. **(C)** Label-free quantification of protein of interest by mass spectrometry. Weighted spectral counts were calculated (means ± SD, *n* = 2) from the analysis of two different pools of eight RIPK3 immunoprecipitates for RIPK3, MLKL, RIPK1, FADD, Caspase 8, HS90B, TRIM21, and PGAM5 observed in IgG control (aIgG) and at times 0 and 3 h post-stimulation. (**) *p* < 0.01.

TRAIL/zVAD/Birinapant (TzB) treatment induced a significant increase in propidium iodide positive cells (60%) at 24 h in HT29 cells. TzB-dependent death was inhibited by both necrostatin-1 (Nec1), a RIPK1 inhibitor (Degterev et al., 2008), and necrosulfosamide (NSA), an inhibitor of RIPK3-MLKL interaction (Sun et al., 2012) (Figure 1A), confirming that TzB-treatment induced necroptosis. Treatment with z-VAD-fmk and Birinapant only was checked not to induce cell death (Supplementary Figure S1A).

In order to investigate TRAIL-induced necrosome timing, cytoplasmic extracts from HT29 cells were subjected to immunoprecipitation using an anti-RIPK1 antibody at different time points after TzB stimulation. Western Blot analysis showed interaction of endogenous RIPK3 with RIPK1 upon TzB-induced necroptosis (Figure 1B). This was confirmed by immunoprecipitation using an anti-RIPK3 antibody, which showed a strong interaction of RIPK1 with RIPK3 at 3 h (Figure 1B). An up-shift of RIPK3 and a smear for RIPK1 were also observed in RIPK1 and RIPK3 immunoprecipitates at 3 h (Figure 1B) suggesting RIPK3 phosphorylation and post-translational modifications of RIPK1 upon TzB-induced necroptosis.

### Mass Spectrometry Analysis of Endogenous TRAIL-Induced RIPK3-Dependent Necrosome Identifies TRIM21 as a new Partner

Immunoprecipitation using anti-RIPK3 antibody was performed to characterize, by mass spectrometry, protein interactors of endogenous RIPK3 at 0 and 3 h post-stimulation with TzB treatment. Based on the model of RIPK3 oligomerization by RIPK1 in response to receptor signaling (Orozco et al., 2014), RIPK3 immunoprecipitates were analyzed by LC-MS/MS for each time point. Proteins identified for each condition were compared by spectral count label-free quantification. We detected the presence of RIPK3 and MLKL peptides at time point 0 h (Figure 1C). RIPK1, FADD and caspase 8 were observed at time 3 h and suggested a RIPK3-dependent necrosome formation upon TzB treatment in HT29 cells. Peptides from two other proteins, HS90B and PGAM5, were also detected in the RIPK3-precipitated complex at time point 3 h (Figure 1C; Supplementary Table S1). These proteins have already been found recruited to the necrosome upon treatment with TNF/Smac mimetic/z-VAD-fmk (Wang et al., 2012; Li et al., 2015). Interestingly, our data identified for the first time the presence of TRIM21 peptides in RIPK3 immunoprecipitate. Spectral count quantification revealed a significant increase of TRIM21 at time point 3 h in comparison with time 0 h (Figure 1C), suggesting increased TRIM21 recruitment in RIPK3-dependent necrosome upon TzB stimulation. None of the identified proteins has been found in the IgG control immunoprecipitate (Figure 1C; Supplementary Table S1, Supplemental Information).

### TRIM21 is Recruited to the Endogenous RIPK3-dependent Necrosome Upon TzB Treatment

**FIGURE 2:**
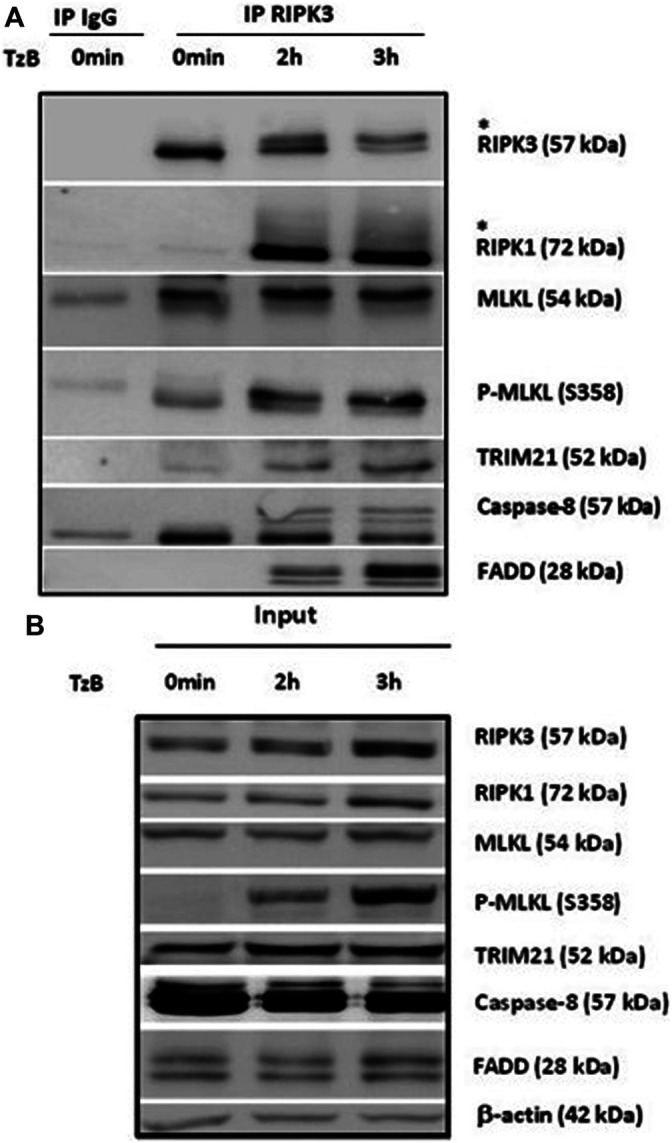
RIPK1, MLKL, P-MLKL, and TRIM21 are recruited to the endogenous RIPK3-dependent necrosome. HT29 cells were treated or not (0 min) with TRAIL-SK (500 ng/ml), z-VAD-fmk (25 µM), and Birinapant (Bp, 1 µM) (TzB) for the indicated times. Four mg of cell lysates were immunoprecipitated (IP) with RIPK3 antibody or control IgG as described in materials and methods. Upper panel: RIPK3 immunocomplexes were analyzed for indicated proteins by immunoblotting. *: an upper band for RIPK3 or a smear for RIPK1. Lower panel: expression of the indicated proteins was analyzed in the total cell lysates (input) by immunoblotting. Anti-human β-actin antibody was used as protein loading control. Representative data of three independent experiments.

To corroborate the recruitment of TRIM21 into the RIPK3-dependent necrosome, HT29 cells were treated with TzB. After 0, 2 or 3 h of treatment, immunoprecipitation using an anti-RIPK3 antibody were conducted. As shown in Figure 2A, low level of TRIM21 was detected at time point 0 h and the amount of TRIM21 increased in a time-dependent manner in the RIPK3-dependent complex upon TzB treatment. This confirmed that TRIM21 is a new partner of the RIPK3-dependent necrosome complex and supported proteomic data (Figure 1C). We also observed an up-shift of RIPK3 after 2 h of treatment (Figure 2A), suggesting RIPK3 phosphorylation as previously shown after TNF/z-VAD-fmk/SM stimulation in HT29 cells (He et al., 2009). In parallel, the phosphorylation of MLKL was evidenced from 2 h to 3 h after TzB treatment as showed by anti-P-MLKL antibody, which was also confirmed in the corresponding total lysates (Figure 2B). Altogether, these data showed for the first time the formation of RIPK3-dependent endogenous necrosome complex upon TzB treatment in HT29 cells containing RIPK1, MLKL, FADD, caspase-8, and TRIM21.

### TRIM21 is a Positive Regulator of TzB-Induced Necroptosis in HT29 and HaCat Cells

**FIGURE 3:**
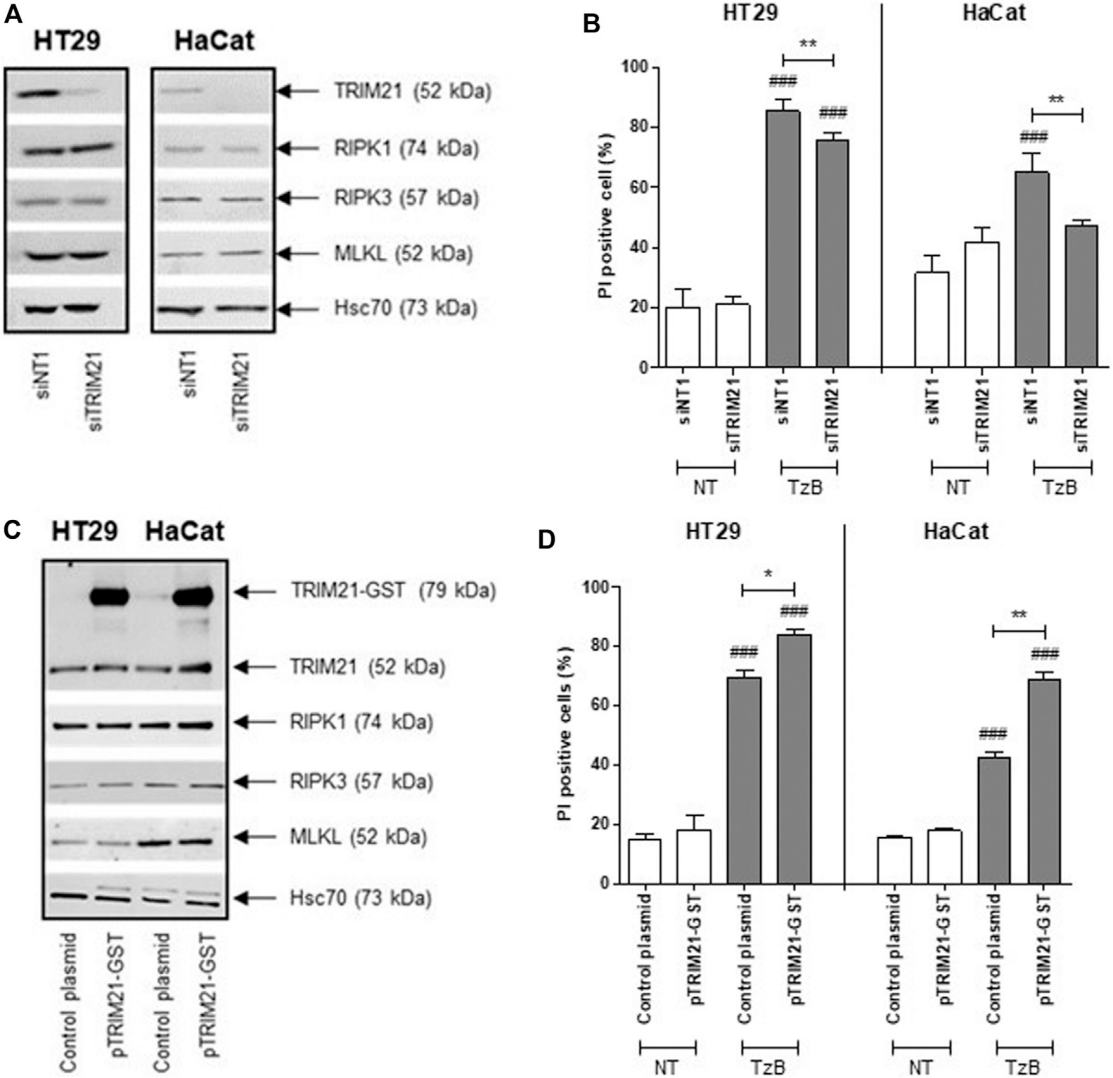
Downregulation or overexpression of TRIM21 expression negatively or positively regulates TRAIL/z-VAD-fmk/Birinapant (TzB)-induced necroptosis, respectively. **(A)** HT29 and HaCat cells were transiently transfected with siTRIM21 or siNT1 (negative control). Immunoblot analysis of TRIM21, RIPK1, RIPK3, and MLKL expressions was carried out 72 h post-transfection. Anti-human Hsc70 antibody was used as protein loading control. Representative data of three independent experiments. **(B)** Forty eight hours after siRNA transfections, HT29, and HaCat cells were treated (in grey) or not (in white) with TRAIL-SK (100 ng/ml), z-VAD-fmk (25 µM) and Birinapant (Bp, 1 µM) (TzB) for 24 h. Percentages of necrotic cells (propidium iodide (PI) positive) were estimated (means ± SD, *n* = 3). **(C)** HT29 and HaCat cells were transiently transfected with pTRIM21-GST or control plasmid (used as a negative control). Immunoblot analysis of TRIM21, RIPK1, RIPK3, and MLKL expressions was carried out 72 h after transfection. Anti-human Hsc70 antibody was used as protein loading control. Representative data of three independent experiments. **(D)** Forty eight hours after transfection, HT29, and HaCat cells were treated (in grey) or not (in white) with TRAIL-SK (100 ng/ml), z-VAD-fmk (25 µM), and Birinapant (Bp, 1 µM) (TzB) for 24 h. Percentages of necrotic cells (propidium iodide (PI) positive) were estimated (means ± SD, *n* = 3). (###), *p* < 0.001 compared treated cells to NT cells; (*), *p* < 0.05, and (**), *p* < 0.01 compared treated conditions.

First, transient transfection with TRIM21 targeted small interference RNA (siRNA), reduced TRIM21 protein expression, but did not alter RIPK1, RIPK3 or MLKL protein expression in HT29 and HaCat cells (Figure 3A). The reduced TRIM21 expression significantly decreased the percentage of necrotic cells induced by treatment with TzB in both HT29 and HaCat cells (Figure 3B). On the other hand, transient transfection with a plasmid coding for TRIM21 tagged with GST (pTRIM21-GST) was used to overexpress TRIM21-GST in both HT29 and HaCat cells without modifying the expressions of endogenous TRIM21, RIPK1, RIPK3 or MLKL (Figure 3C). TRIM21 overexpression significantly sensitized both HT29 and HaCat cells to TzB treatment by increasing the percentage of necrotic cells (Figure 3D). These data showed that TRIM21 is a new positive modulator of TRAIL-induced necroptosis.

### CRISPR/Cas9-Mediated TRIM21 Knockout in HT29 Cells Confirms that TRIM21 is a Positive and Specific Modulator of TzB-Induced Necroptosis.

**FIGURE 4:**
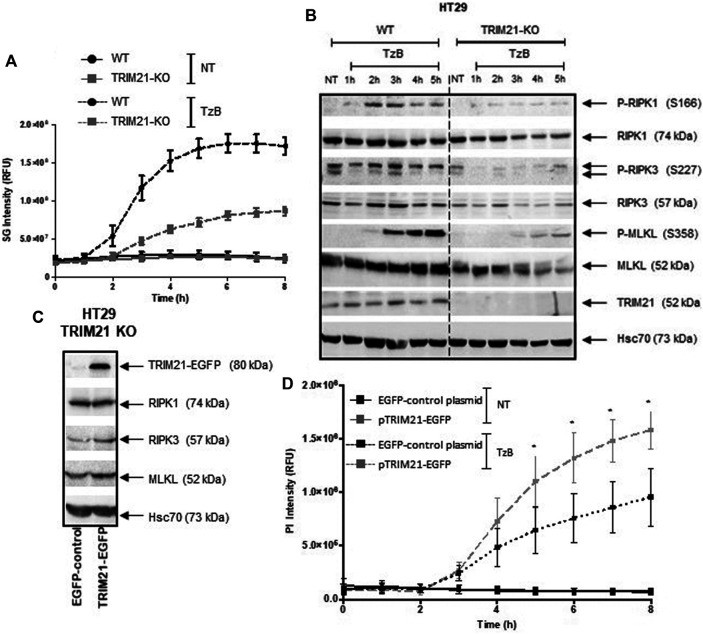
TRIM21-KO HT29 cells are more resistant to TRAIL/z-VAD-fmk/Birinapant (TzB)-induced necroptosis and their sensitivity can be rescued by TRIM21 re-expression. **(A)** WT (black lines) or TRIM21-KO (grey lines) HT29 cells were treated (dashed lines) or not (solid lines) with TRAIL-SK (100 ng/ml), z-VAD-fmk (25 µM), and Birinapant (Bp, 1 µM) (TzB) during 8 h. Necroptosis was evaluated by measuring SYTOX™ Green (SG) intensity (RFU) as described in materials and methods. Representative data of three independent experiments. **(B)** WT and TRIM21-KO HT29 cells were treated with TzB during the indicated times. Immunoblot analysis of TRIM21, RIPK1, RIPK3, MLKL, and phosphorylated forms of RIPK1, RIPK3, and MLKL were evaluated. Anti-human Hsc70 antibody was used as protein loading control. Representative data of three independent experiments. **(C)** TRIM21-KO HT29 cells were transfected with EGFP-control plasmid or TRIM21-EGFP plasmid for 48 h and approximately 40% of cells were GFP positive for each transfection conditions. Immunoblot analysis of TRIM21, RIPK1, RIPK3 and, MLKL expressions was carried out 48 h after transfection. Anti-human Hsc70 antibody was used as protein loading control. **(D)** After transfection with the EGFP-control plasmid (black lines) or with the pTRIM21-EGFP (grey lines), cells were treated or not (NT, solid lines) with TRAIL-SK (100 ng/ml), z-VAD-fmk (25 µM), and Birinapant (Bp, 1 µM) (TzB, dashed lines) during 8 h. Necroptosis was evaluated by measuring propidium iodide (PI) intensity (RFU) as described in materials and methods (means ± SEM, *n* = 3). (*), *p* < 0.05 compared treated conditions.

Furthermore, human HT29 cells were transfected with two distinct CRISPR-Cas9 gRNA-plasmids to abrogate TRIM21 expression. Western blot analysis validated the complete loss of TRIM21 expression in TRIM21-KO HT29 cells compared to wild-type (WT) HT29 cells. No change in expression of RIPK1, RIPK3 or MLKL was observed in TRIM21-KO HT29 cells (Supplementary Figure S1B, Supplemental Information). To further characterize the impact of TRIM21 loss in HT29 cells, cell death by TzB-induced necroptosis was followed over time in both WT and TRIM21-KO HT29 cells using SYTOX^TM^ Green reagent staining (Figure 4A). While WT HT29 cells began to die by necroptosis at 2 h after TzB treatment, cell death signal was delayed for TRIM21-KO HT29 cells and started to increase at 3 h to reach a maximum intensity 3 times lower. In parallel, Western blot analysis showed that, after TzB treatment, not only a lower phosphorylation state of RIPK1 and MLKL, but also a delay in the onset of MLKL phosphorylation took place in TRIM21-KO HT29 cells when compared to what happened in WT HT29 cells (Figure 4B). In fact, after TzB treatment, phosphorylation of MLKL was detected from 2 to 5 h in WT HT29 cells, whereas only low level of P-MLKL was observed from 3 to 5 h in TRIM21-KO HT29 cells. Moreover, RIPK3 levels were upregulated in WT HT29 cells in the first 3 h after TzB treatment. Such an increase in the expression of RIPK3 has already been described upon TNF-induced necroptosis (Afonso et al., 2015). In contrast, upon TzB treatment, RIPK3 levels varied only slightly in TRIM21-KO HT29 cells that probably contributed to limit cell death (Figure 4B). To verify that this loss of sensitivity to TzB-induced necroptosis observed in TRIM21-KO HT29 cells was specifically related to the absence of TRIM21, rescue experiments were conducted. Thus, transient transfection of TRIM21-KO HT29 cells with the pTRIM21-EGFP expression vector induced TRIM21-EGFP expression (Figure 4C) and a greater sensitivity to TzB-induced necroptosis followed over-time (Figure 4D). These investigations reinforced the data obtained above with the siRNA-based approach (Figures 3A,B), further validating TRIM21 as a positive modulator of this cell death pathway. Moreover, the loss of TRIM21 expression only affected the sensitivity of TRIM21-KO HT29 cells to TzB-induced necroptosis (Supplementary Figure S1E, Supplemental Information) but did not affect the sensitivity of TRIM21-KO HT29 cells to apoptosis induced by the combination of Birinapant (Bp) with TRAIL, TNF or Ig-FasL (Supplementary Figure S1D, Supplemental Information), nor to necroptosis induced by z-VAD-fmk/Bp with TNF or Ig-FasL (Supplementary Figure S1E, Supplemental Information). Altogether, these data suggest that TRIM21 is a positive and specific regulator of TzB necroptotic cell death pathway.

## Discussion

TRAIL is a potential anticancer drug that induces apoptosis or necroptosis depending on cell conditions (Vasilikos et al., 2017). A lot of hope has been put on TRAIL to fight carcinomas, but cancer cells have developed several molecular mechanisms of resistance to TRAIL-induced apoptosis (Newsom-Davis et al., 2009). A strategy to overcome apoptosis resistance is to induce necroptosis, a backup cell death pathway (Han et al., 2011).

In this work, TRAIL treatment in combination with z-VAD-fmk (a pan-caspase inhibitor) and Birinapant (a pan-IAP inhibitor) (TzB) was used to induce necroptosis in HT29 cells as previously described by He et al. in 2009. It is important to note that RIPK3 is not expressed in most cancer cell lines due to methylation-dependent regulations, however HaCat cells were described to be sensitive to TzB-induced necroptosis (Karl et al., 2014). This necroptotic death pathway may be useful to characterize protein interactors into the necrosome. First, experiments of co-immunoprecipitation of RIPK3 with RIPK1 demonstrated that both of these serine-threonine kinases bind to each other upon TzB-induced necroptosis in HT29 cells with a strong interaction of RIPK1 with RIPK3 at 3 h post-treatment. This time point was used thereafter to characterize by proteomics the protein interactors of the endogenous necrosome induced by TzB in HT29 cells and immunoprecipitated with an anti-RIPK3 antibody (Orozco et al., 2014). In fact, in the past years, the protein composition of the necrosome has mainly been studied in response to TNF. Notably, several RIPK3 interactors in TNF-induced necroptosis have been identified with exogenous expression of tagged-RIPK3 in different cell lines (HEK293, NIH3T3 or L929-RIPK3^−/−^ cells) (Bouwmeester et al., 2004; Zhang et al., 2009; Chen et al., 2015). Here, we characterized for the first time, by mass spectrometry, a novel protein in the endogenous necrosome complex immunoprecipitated with an anti-RIPK3 antibody upon TzB treatment. Three hours post-stimulation with TzB, known components of necrosome complex such as RIPK1, FADD, caspase-8, and MLKL were found in the anti-RIPK3 immunoprecipitate. More interestingly, a new protein, TRIM21, was also put in evidence in the RIPK3-dependent necrosome. TRIM21 (also known as Ro52) belongs to the RING E3 ligases family of tripartite motif (TRIM) proteins (Tomar and Singh, 2015) involved in ubiquitination processes of various proteins implied either in the cell cycle (Sabile et al., 2006), in the antiviral immune response (Young et al., 2011) or in apoptosis (Jauharoh et al., 2012; Zhang et al., 2012).

Among others interactors found in the endogenous RIPK3-dependent necrosome, phosphoglycerate mutase 5 (PGAM5) and HS90B (Heat shock protein 90-beta) peptides were detected in RIPK3 immunoprecipitate at time point 3 h. A study showed that PGAM5 may play a role in TNF/Smac mimetic/z-VAD-fmk-induced necroptosis via its recruitment to the necrosome by RIPK3, leading to mitochondrial fragmentation and ROS production (Wang et al., 2012). HSP90 and its co-chaperone CDC37 were characterized in HA-Flag-tagged RIPK3 after TNF/Smac mimetic/z-VAD-fmk in HT29 cells and were required for RIPK3 activation and necroptosis induction (Li et al., 2015). Nonetheless, further studies are needed for a better understanding of the role of PGAM5 and HS90B in TRAIL/z-VAD-fmk/Birinapant-induced necroptosis.

The recruitment of TRIM21 into the RIPK3-dependent necrosome was confirmed in a kinetic experiment of TzB-induced necroptosis. Besides RIPK1, FADD, caspase-8 and MLKL, TRIM21 was detected in the RIPK3-dependent necrosome. A low level of TRIM21 was found in basal conditions (without stimulation) and increased from 2 to 3 h post-treatment with TzB, confirming the recruitment of TRIM21 into the necrosome.

To further characterize the role of TRIM21 in TzB-induced necroptosis, downregulation by RNA interference and overexpression experiments of this E3 ubiquitin-protein ligase were used in two human cell lines HT29 (colon cancer cells) and HaCat (immortalized keratinocyte cells). Both cell lines express RIPK1, RIPK3 and MLKL, and necroptotic stimulus, such as TzB, could induce cell death. Downregulation or overexpression of TRIM21 in both cell lines showed a decrease or an increase in necroptosis induction, respectively, suggesting that TRIM21 is a positive modulator of TzB-induced necroptosis. This role of TRIM21 was confirmed in TRIM21 knockout HT29 cells (TRIM21-KO cells) obtained by CRISPR/Cas9, which are more resistant to TzB-induced necroptosis in comparison to WT HT29 cells. It has to be noticed that the invalidation of TRIM21 expression by CRISPR-Cas9 or alteration of TRIM21 by RNA interference did not modify the expression of death receptors (DR4 and DR5) in HT29 cells (Supplementary Figure S1C, Supplemental Information). Furthermore, transfection of human TRIM21 cDNA in TRIM21-KO cells rendered these cells more responsive to TzB-induced necroptosis and rescued the sensitive phenotype of WT HT29 cells.

Finally, in a time course experiment, the expression of TRIM21 was positively correlated to the phosphorylation state of two key proteins RIPK1 and MLKL involved in the induction of necroptosis induced by TRAIL. Altogether, our data showed that TRIM21 is a positive modulator of TRAIL-induced necroptosis.

Since TRIM21 has previously been shown to sensitize H1299 cells to TRAIL-induced apoptosis (Gao et al., 2016), we compared the sensitivity of TRIM21 WT and TRIM21-KO cells to apoptosis or necroptosis induced by several death ligands (TRAIL, FasL or TNF). Unexpectedly, TRIM21-KO cells appear to be only resistant to TRAIL/z-VAD-fmk/Bp-induced necroptosis suggesting that TRIM21 is a specific and positive modulator of this necroptotic pathway.

Besides a role for TRIM21 in many cellular processes such as cell proliferation, apoptosis, and innate and adaptive immunity, several studies have shown that TRIM21 is involved in the progression of human cancers. Decreased expression of TRIM21 has been found in hepatocellular carcinoma, diffuse large B-cell lymphoma and breast cancer, and was associated with a poor prognosis and enhanced proliferation capacity of cancer cells *in vitro* and tumor growth *in vivo* (Brauner et al., 2015; Ding et al., 2015; Zhou et al., 2018). Our present data also suggest that cancer with low TRIM21 expression could be more resistant to TRAIL-induced necroptosis, a backup cell death pathway that can be triggered when apoptosis cannot occur. In this context, novel therapeutic approaches to restore TRIM21 expression could be clinically beneficial.

In conclusion, our works describe for the first time the characterization by mass spectrometry of proteins that are co-immunoprecipitated with endogenous RIPK3 upon TRAIL/zVAD/Birinapant-induced necroptosis. Among known proteins to be associated with TRAIL-induced necrosome such as RIPK1, MLKL, FADD, and caspase-8, a new partner was discovered, the E3 ubiquitin ligase TRIM21 which positively and specifically modulates TRAIL-induced necroptosis. Taken together, these results provide a new insight in the TRAIL necroptotic pathway which could be useful to treat apoptosis resistant cancer.

## Data Availability

The datasets presented in this study can be found in online repositories. The names of the repository/repositories and accession number(s) can be found below: http://www.proteomexchange.org/, PXD003383.
