# Supplementary material for: TRIM21, a New Component of the TRAIL-Induced Endogenous Necrosome Complex
Source: Front Mol Biosci. 2021 Apr 15;8:645134. doi: 10.3389/fmolb.2021.645134 (PMC8082149; doi:10.3389/fmolb.2021.645134)
Supplement: Supplementary file 1 [file datasheet1.pdf]

Supplementary Material

Fig. S1 The combination of z-VAD-fmk and Birinapant does not decrease cell viability in HT29 or HaCat cells. Knock-out of TRIM21 expression in HT29 cells by CRISPR/Cas9 only modifies cell sensitivity to TzB-induced necroptosis. **(A)** HT29 cells (black columns) and HaCat cells (white columns) are treated or not (NT) with z-VAD-fmk (25 µM) and Birinapant (Bp, 1 µM) for 24 h. Cell viability (%) was estimated by MTS assay (mean ± SD, n=3). **(B)** TRIM21-KO HT29 cell line was obtained by CRISPR-Cas9 as described in materials and methods. Immunoblot analysis of TRIM21, RIPK1, RIPK3 and MLKL expressions was performed. Anti-human Hsc70 antibody was used as protein loading control. Representative data of three independent experiments. **(C)** The invalidation of TRIM21 expression by CRISPR-Cas9 or alteration of TRIM21 by RNA interference does not modify the expression of death receptors (DR4 and DR5) in HT29 cells. **(D)**WT (black dashed lines) and TRIM21-KO (grey dashed lines) HT29 cells were treated with increased concentrations of TRAIL-SK (a), TNF-α (b) or Ig-FasL (c) in presence of Birinapant (Bp, 1 µM) during 24 h. Cell viability (%) was estimated by MTS assay (mean ± SD, n=3). **(E)** WT (black dashed line) and TRIM21-KO (grey dashed lines) HT29 cells were treated with increased concentrations of TRAIL-SK (a), TNF-α (b) or Ig-FasL (c) in presence of z-VAD-fmk (25 µM) and Birinapant (Bp, 1 µM) during 24 h. Cell viability (%) was estimated by MTS assay (mean ± SD, n=3). (**), *P*<0.01 compared treated conditions.

Table S1 Protein table: identification of proteins of interest with the Uniprot Accession and the protein description; the protein identification scores, the % sequence coverage, the number of identified peptides, the number of peptide spectrum matches (PSM), the weighted spectral count (with the average value and the standard deviation) obtained for the different experiments realized in duplicates (Exp.1 and 2): aIgG control, 0 h and 3 h post-stimulation. (nd= non detected protein). Peptide table: identification of peptides corresponding to the proteins of interest with the Uniprot Accession and the protein description; the peptide sequence, the modifications, the ion score, the experimental m/z and the charge, the calculated mass of the peptide, the delta mass shift, the number of miscleavages, the number of fragment ions matches obtained for the different experiments realized in duplicates (Exp.1 and 2): aIgG control, 0 h and 3 h post-stimulation.

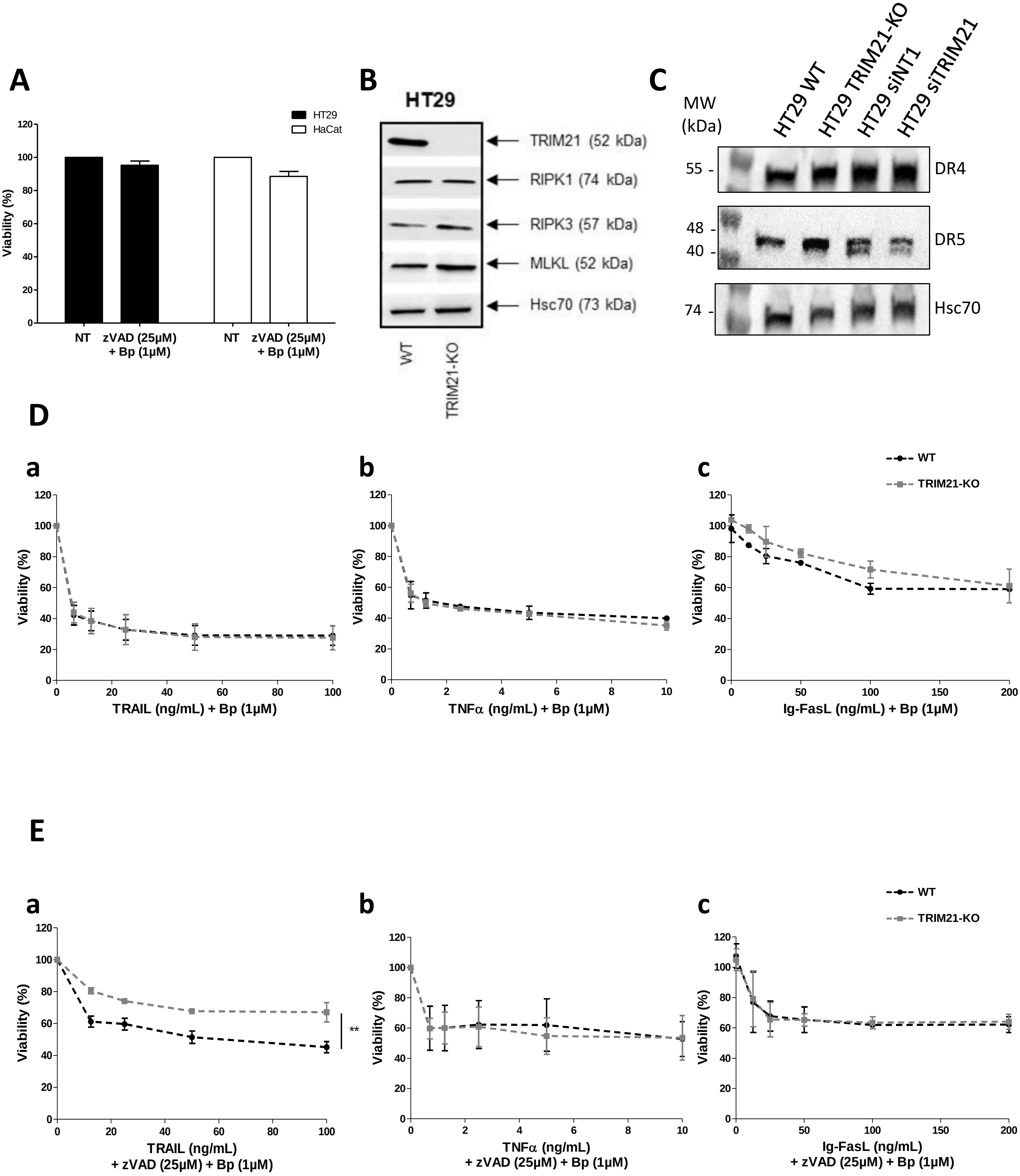
A
HT29
HaCat
Viability (%)
NT
zVAD (25µM) + Bp (1µM)
B
HT29
TRIM21 (52 kDa)
RIPK1 (74 kDa)
RIPK3 (57 kDa)
MLKL (52 kDa)
Hsc70 (73 kDa)
WT
TRIM21-KO
C
MW (kDa)
HT29 WT
HT29 TRIM21-KO
HT29 siNT1
HT29 siTRIM21
DR4
DR5
Hsc70
D
a
b
c
WT
TRIM21-KO
TRAIL (ng/mL) + Bp (1µM)
TNFα (ng/mL) + Bp (1µM)
Ig-FasL (ng/mL) + Bp (1µM)
E
TRAIL (ng/mL) + zVAD (25µM) + Bp (1µM)
TNFα (ng/mL) + zVAD (25µM) + Bp (1µM)
Ig-FasL (ng/mL) + zVAD (25µM) + Bp (1µM)
**

Figure S1

Protein Table

Uniprot Accession	Protein Description
sp|P08238|HS90B_HUMAN	Heat shock protein HSP 90-beta OS=Homo sapiens GN=HSP90AB1 PE=1 SV=4
sp|P19474|RO52_HUMAN	E3 ubiquitin-protein ligase TRIM21 OS=Homo sapiens GN=TRIM21 PE=1 SV=1
sp|Q12933|TRAF2_HUMAN	TNF receptor-associated factor 2 OS=Homo sapiens GN=TRAF2 PE=1 SV=2
sp|Q13158|FADD_HUMAN	FAS-associated death domain protein OS=Homo sapiens GN=FADD PE=1 SV=1
sp|Q13546|RIPK1_HUMAN	Receptor-interacting serine/threonine-protein kinase 1 OS=Homo sapiens GN=RIPK1 PE=1 SV=3
sp|Q14790|CASP8_HUMAN	Caspase-8 OS=Homo sapiens GN=CASP8 PE=1 SV=1
sp|Q15628|TRADD_HUMAN	Tumor necrosis factor receptor type 1-associated DEATH domain protein OS=Homo sapiens GN=TRADD PE=1 SV=2
sp|Q8NB16|MLKL_HUMAN	Mixed lineage kinase domain-like protein OS=Homo sapiens GN=MLKL PE=1 SV=1
sp|Q96HS1|PGAM5_HUMAN	Serine/threonine-protein phosphatase PGAM5, mitochondrial OS=Homo sapiens GN=PGAM5 PE=1 SV=2
sp|Q9Y572|RIPK3_HUMAN	Receptor-interacting serine/threonine-protein kinase 3 OS=Homo sapiens GN=RIPK3 PE=1 SV=2

	Score						% Sequence coverage						Number of distinct peptides					
	IgG		0 h		3 h		IgG		0 h		3 h		IgG		0 h		3 h	
Uniprot Accession	Exp. 1	Exp. 2	Exp. 1	Exp. 2	Exp. 1	Exp. 2	Exp. 1	Exp. 2	Exp. 1	Exp. 2	Exp. 1	Exp. 2	Exp. 1	Exp. 2	Exp. 1	Exp. 2	Exp. 1	Exp. 2
sp|P08238|HS90B_HUMAN	nd	nd	nd	nd	63,51	102	nd	nd	nd	nd	3,2	2,8	nd	nd	nd	nd	2	2
sp|P19474|RO52_HUMAN	nd	nd	72,11	105	256,2	679,2	nd	nd	9,1	5,9	26,3	39,2	nd	nd	3	2	8	15
sp|Q12933|TRAF2_HUMAN	nd	nd	nd	nd	nd	111	nd	nd	nd	nd	nd	6,4	nd	nd	nd	nd	nd	3
sp|Q13158|FADD_HUMAN	nd	nd	nd	nd	36,34	194,2	nd	nd	nd	nd	8,2	28,8	nd	nd	nd	nd	1	4
sp|Q13546|RIPK1_HUMAN	nd	nd	nd	nd	124	561,8	nd	nd	nd	nd	4,8	19,8	nd	nd	nd	nd	2	11
sp|Q14790|CASP8_HUMAN	nd	nd	nd	nd	224,7	305,6	nd	nd	nd	nd	10,6	13,6	nd	nd	nd	nd	4	6
sp|Q15628|TRADD_HUMAN	nd	nd	nd	nd	nd	71.69	nd	nd	nd	nd	nd	7,7	nd	nd	nd	nd	nd	2
sp|Q8NB16|MLKL_HUMAN	nd	nd	38,69	nd	266,6	430	nd	nd	5,3	nd	21,7	25,7	nd	nd	1	nd	7	9
sp|Q96HS1|PGAM5_HUMAN	nd	nd	nd	nd	88,18	191,1	nd	nd	nd	nd	16,3	14,9	nd	nd	nd	nd	3	4
sp|Q9Y572|RIPK3_HUMAN	nd	nd	1111	644,6	1121	572	nd	nd	67,6	39	62,5	34,6	nd	nd	24	13	24	12

	Number of PSM						Weighted Spectral Count											
	IgG		0 h		3 h		IgG		0 h				3 h			
**Uniprot Accession**	**Exp. 1**	**Exp. 2**	**Exp. 1**	**Exp. 2**	**Exp. 1**	**Exp. 2**	**Exp. 1**	**Exp. 2**	**Exp. 1**	**Exp. 2**	**Average**	**ard Dev**	**Exp. 1**	**Exp. 2**	**Av.**	**SD**
sp|P08238|HS90B_HUMAN	nd	nd	nd	nd	2	2	nd	nd	nd	nd	**nd**	**nd**	1,67	1,67	**1,67**	**0,00**
sp|P19474|RO52_HUMAN	nd	nd	3	2	9	16	nd	nd	3	2	**2,5**	**0,71**	9	16	**12,5**	**4,95**
sp|Q12933|TRAF2_HUMAN	nd	nd	nd	nd	nd	4	nd	nd	nd	nd	**nd**	**nd**	nd	4	**2**	**2,83**
sp|Q13158|FADD_HUMAN	nd	nd	nd	nd	1	4	nd	nd	nd	nd	**nd**	**nd**	1	4	**2,5**	**2,12**
sp|Q13546|RIPK1_HUMAN	nd	nd	nd	nd	2	11	nd	nd	nd	nd	**nd**	**nd**	2	11	**6,5**	**6,36**
sp|Q14790|CASP8_HUMAN	nd	nd	nd	nd	4	6	nd	nd	nd	nd	**nd**	**nd**	4	6	**5**	**1,41**
sp|Q15628|TRADD_HUMAN	nd	nd	nd	nd	nd	2	nd	nd	nd	nd	**nd**	**nd**	nd	2	**1**	**1,41**
sp|Q8NB16|MLKL_HUMAN	nd	nd	1	nd	7	10	nd	nd	1	nd	**0,5**	**0,71**	7	10	**8,5**	**2,12**
sp|Q96HS1|PGAM5_HUMAN	nd	nd	nd	nd	3	6	nd	nd	nd	nd	**nd**	**nd**	3	6	**4,5**	**2,12**
sp|Q9Y572|RIPK3_HUMAN	nd	nd	32	15	31	14	nd	nd	32	15	**23,5**	**12,02**	31	14	**22,50**	**12,02**

(Av. : Average; SD: Standard Deviation)

Peptide Table

Uniprot accession	description
P08238	Heat shock protein HSP 90-beta OS=Homo sapiens GN=HSP90AB1 PE=1 SV=4
P08238	Heat shock protein HSP 90-beta OS=Homo sapiens GN=HSP90AB1 PE=1 SV=4
P08238	Heat shock protein HSP 90-beta OS=Homo sapiens GN=HSP90AB1 PE=1 SV=4
P08238	Heat shock protein HSP 90-beta OS=Homo sapiens GN=HSP90AB1 PE=1 SV=4
P19474	E3 ubiquitin-protein ligase TRIM21 OS=Homo sapiens GN=TRIM21 PE=1 SV=1
P19474	E3 ubiquitin-protein ligase TRIM21 OS=Homo sapiens GN=TRIM21 PE=1 SV=1
P19474	E3 ubiquitin-protein ligase TRIM21 OS=Homo sapiens GN=TRIM21 PE=1 SV=1
P19474	E3 ubiquitin-protein ligase TRIM21 OS=Homo sapiens GN=TRIM21 PE=1 SV=1
P19474	E3 ubiquitin-protein ligase TRIM21 OS=Homo sapiens GN=TRIM21 PE=1 SV=1
P19474	E3 ubiquitin-protein ligase TRIM21 OS=Homo sapiens GN=TRIM21 PE=1 SV=1
P19474	E3 ubiquitin-protein ligase TRIM21 OS=Homo sapiens GN=TRIM21 PE=1 SV=1
P19474	E3 ubiquitin-protein ligase TRIM21 OS=Homo sapiens GN=TRIM21 PE=1 SV=1
P19474	E3 ubiquitin-protein ligase TRIM21 OS=Homo sapiens GN=TRIM21 PE=1 SV=1
P19474	E3 ubiquitin-protein ligase TRIM21 OS=Homo sapiens GN=TRIM21 PE=1 SV=1
P19474	E3 ubiquitin-protein ligase TRIM21 OS=Homo sapiens GN=TRIM21 PE=1 SV=1
P19474	E3 ubiquitin-protein ligase TRIM21 OS=Homo sapiens GN=TRIM21 PE=1 SV=1
P19474	E3 ubiquitin-protein ligase TRIM21 OS=Homo sapiens GN=TRIM21 PE=1 SV=1
P19474	E3 ubiquitin-protein ligase TRIM21 OS=Homo sapiens GN=TRIM21 PE=1 SV=1
P19474	E3 ubiquitin-protein ligase TRIM21 OS=Homo sapiens GN=TRIM21 PE=1 SV=1
P19474	E3 ubiquitin-protein ligase TRIM21 OS=Homo sapiens GN=TRIM21 PE=1 SV=1
P19474	E3 ubiquitin-protein ligase TRIM21 OS=Homo sapiens GN=TRIM21 PE=1 SV=1
P19474	E3 ubiquitin-protein ligase TRIM21 OS=Homo sapiens GN=TRIM21 PE=1 SV=1
P19474	E3 ubiquitin-protein ligase TRIM21 OS=Homo sapiens GN=TRIM21 PE=1 SV=1
P19474	E3 ubiquitin-protein ligase TRIM21 OS=Homo sapiens GN=TRIM21 PE=1 SV=1
P19474	E3 ubiquitin-protein ligase TRIM21 OS=Homo sapiens GN=TRIM21 PE=1 SV=1
P19474	E3 ubiquitin-protein ligase TRIM21 OS=Homo sapiens GN=TRIM21 PE=1 SV=1
P19474	E3 ubiquitin-protein ligase TRIM21 OS=Homo sapiens GN=TRIM21 PE=1 SV=1
P19474	E3 ubiquitin-protein ligase TRIM21 OS=Homo sapiens GN=TRIM21 PE=1 SV=1
P19474	E3 ubiquitin-protein ligase TRIM21 OS=Homo sapiens GN=TRIM21 PE=1 SV=1

Uniprot accession	description
P19474	E3 ubiquitin-protein ligase TRIM21 OS=Homo sapiens GN=TRIM21 PE=1 SV=1
P19474	E3 ubiquitin-protein ligase TRIM21 OS=Homo sapiens GN=TRIM21 PE=1 SV=1
P19474	E3 ubiquitin-protein ligase TRIM21 OS=Homo sapiens GN=TRIM21 PE=1 SV=1
Q12933	TNF receptor-associated factor 2 OS=Homo sapiens GN=TRAF2 PE=1 SV=2
Q12933	TNF receptor-associated factor 2 OS=Homo sapiens GN=TRAF2 PE=1 SV=2
Q12933	TNF receptor-associated factor 2 OS=Homo sapiens GN=TRAF2 PE=1 SV=2
Q13158	FAS-associated death domain protein OS=Homo sapiens GN=FADD PE=1 SV=1
Q13158	FAS-associated death domain protein OS=Homo sapiens GN=FADD PE=1 SV=1
Q13158	FAS-associated death domain protein OS=Homo sapiens GN=FADD PE=1 SV=1
Q13158	FAS-associated death domain protein OS=Homo sapiens GN=FADD PE=1 SV=1
Q13158	FAS-associated death domain protein OS=Homo sapiens GN=FADD PE=1 SV=1
Q13546	Receptor-interacting serine/threonine-protein kinase 1 OS=Homo sapiens GN=RIPK1 PE=1 SV=3
Q13546	Receptor-interacting serine/threonine-protein kinase 1 OS=Homo sapiens GN=RIPK1 PE=1 SV=3
Q13546	Receptor-interacting serine/threonine-protein kinase 1 OS=Homo sapiens GN=RIPK1 PE=1 SV=3
Q13546	Receptor-interacting serine/threonine-protein kinase 1 OS=Homo sapiens GN=RIPK1 PE=1 SV=3
Q13546	Receptor-interacting serine/threonine-protein kinase 1 OS=Homo sapiens GN=RIPK1 PE=1 SV=3
Q13546	Receptor-interacting serine/threonine-protein kinase 1 OS=Homo sapiens GN=RIPK1 PE=1 SV=3
Q13546	Receptor-interacting serine/threonine-protein kinase 1 OS=Homo sapiens GN=RIPK1 PE=1 SV=3
Q13546	Receptor-interacting serine/threonine-protein kinase 1 OS=Homo sapiens GN=RIPK1 PE=1 SV=3
Q13546	Receptor-interacting serine/threonine-protein kinase 1 OS=Homo sapiens GN=RIPK1 PE=1 SV=3
Q13546	Receptor-interacting serine/threonine-protein kinase 1 OS=Homo sapiens GN=RIPK1 PE=1 SV=3
Q13546	Receptor-interacting serine/threonine-protein kinase 1 OS=Homo sapiens GN=RIPK1 PE=1 SV=3
Q13546	Receptor-interacting serine/threonine-protein kinase 1 OS=Homo sapiens GN=RIPK1 PE=1 SV=3
Q13546	Receptor-interacting serine/threonine-protein kinase 1 OS=Homo sapiens GN=RIPK1 PE=1 SV=3
Q14790	Caspase-8 OS=Homo sapiens GN=CASP8 PE=1 SV=1
Q14790	Caspase-8 OS=Homo sapiens GN=CASP8 PE=1 SV=1
Q14790	Caspase-8 OS=Homo sapiens GN=CASP8 PE=1 SV=1
Q14790	Caspase-8 OS=Homo sapiens GN=CASP8 PE=1 SV=1
Q14790	Caspase-8 OS=Homo sapiens GN=CASP8 PE=1 SV=1
Q14790	Caspase-8 OS=Homo sapiens GN=CASP8 PE=1 SV=1
Q14790	Caspase-8 OS=Homo sapiens GN=CASP8 PE=1 SV=1

Uniprot accession	description
Q14790	Caspase-8 OS=Homo sapiens GN=CASP8 PE=1 SV=1
Q14790	Caspase-8 OS=Homo sapiens GN=CASP8 PE=1 SV=1
Q14790	Caspase-8 OS=Homo sapiens GN=CASP8 PE=1 SV=1
Q15628	Tumor necrosis factor receptor type 1-associated DEATH domain protein OS=Homo sapiens GN=TRADD PE=1 SV=2
Q15628	Tumor necrosis factor receptor type 1-associated DEATH domain protein OS=Homo sapiens GN=TRADD PE=1 SV=2
Q8NB16	Mixed lineage kinase domain-like protein OS=Homo sapiens GN=MLKL PE=1 SV=1
Q8NB16	Mixed lineage kinase domain-like protein OS=Homo sapiens GN=MLKL PE=1 SV=1
Q8NB16	Mixed lineage kinase domain-like protein OS=Homo sapiens GN=MLKL PE=1 SV=1
Q8NB16	Mixed lineage kinase domain-like protein OS=Homo sapiens GN=MLKL PE=1 SV=1
Q8NB16	Mixed lineage kinase domain-like protein OS=Homo sapiens GN=MLKL PE=1 SV=1
Q8NB16	Mixed lineage kinase domain-like protein OS=Homo sapiens GN=MLKL PE=1 SV=1
Q8NB16	Mixed lineage kinase domain-like protein OS=Homo sapiens GN=MLKL PE=1 SV=1
Q8NB16	Mixed lineage kinase domain-like protein OS=Homo sapiens GN=MLKL PE=1 SV=1
Q8NB16	Mixed lineage kinase domain-like protein OS=Homo sapiens GN=MLKL PE=1 SV=1
Q8NB16	Mixed lineage kinase domain-like protein OS=Homo sapiens GN=MLKL PE=1 SV=1
Q8NB16	Mixed lineage kinase domain-like protein OS=Homo sapiens GN=MLKL PE=1 SV=1
Q8NB16	Mixed lineage kinase domain-like protein OS=Homo sapiens GN=MLKL PE=1 SV=1
Q8NB16	Mixed lineage kinase domain-like protein OS=Homo sapiens GN=MLKL PE=1 SV=1
Q8NB16	Mixed lineage kinase domain-like protein OS=Homo sapiens GN=MLKL PE=1 SV=1
Q8NB16	Mixed lineage kinase domain-like protein OS=Homo sapiens GN=MLKL PE=1 SV=1
Q8NB16	Mixed lineage kinase domain-like protein OS=Homo sapiens GN=MLKL PE=1 SV=1
Q8NB16	Mixed lineage kinase domain-like protein OS=Homo sapiens GN=MLKL PE=1 SV=1
Q96HS1	Serine/threonine-protein phosphatase PGAM5, mitochondrial OS=Homo sapiens GN=PGAM5 PE=1 SV=2
Q96HS1	Serine/threonine-protein phosphatase PGAM5, mitochondrial OS=Homo sapiens GN=PGAM5 PE=1 SV=2
Q96HS1	Serine/threonine-protein phosphatase PGAM5, mitochondrial OS=Homo sapiens GN=PGAM5 PE=1 SV=2
Q96HS1	Serine/threonine-protein phosphatase PGAM5, mitochondrial OS=Homo sapiens GN=PGAM5 PE=1 SV=2
Q96HS1	Serine/threonine-protein phosphatase PGAM5, mitochondrial OS=Homo sapiens GN=PGAM5 PE=1 SV=2
Q96HS1	Serine/threonine-protein phosphatase PGAM5, mitochondrial OS=Homo sapiens GN=PGAM5 PE=1 SV=2
Q96HS1	Serine/threonine-protein phosphatase PGAM5, mitochondrial OS=Homo sapiens GN=PGAM5 PE=1 SV=2
Q9Y572	Receptor-interacting serine/threonine-protein kinase 3 OS=Homo sapiens GN=RIPK3 PE=1 SV=2
Q9Y572	Receptor-interacting serine/threonine-protein kinase 3 OS=Homo sapiens GN=RIPK3 PE=1 SV=2

Uniprot accession	description
Q9Y572	Receptor-interacting serine/threonine-protein kinase 3 OS=Homo sapiens GN=RIPK3 PE=1 SV=2
Q9Y572	Receptor-interacting serine/threonine-protein kinase 3 OS=Homo sapiens GN=RIPK3 PE=1 SV=2
Q9Y572	Receptor-interacting serine/threonine-protein kinase 3 OS=Homo sapiens GN=RIPK3 PE=1 SV=2
Q9Y572	Receptor-interacting serine/threonine-protein kinase 3 OS=Homo sapiens GN=RIPK3 PE=1 SV=2
Q9Y572	Receptor-interacting serine/threonine-protein kinase 3 OS=Homo sapiens GN=RIPK3 PE=1 SV=2
Q9Y572	Receptor-interacting serine/threonine-protein kinase 3 OS=Homo sapiens GN=RIPK3 PE=1 SV=2
Q9Y572	Receptor-interacting serine/threonine-protein kinase 3 OS=Homo sapiens GN=RIPK3 PE=1 SV=2
Q9Y572	Receptor-interacting serine/threonine-protein kinase 3 OS=Homo sapiens GN=RIPK3 PE=1 SV=2
Q9Y572	Receptor-interacting serine/threonine-protein kinase 3 OS=Homo sapiens GN=RIPK3 PE=1 SV=2
Q9Y572	Receptor-interacting serine/threonine-protein kinase 3 OS=Homo sapiens GN=RIPK3 PE=1 SV=2
Q9Y572	Receptor-interacting serine/threonine-protein kinase 3 OS=Homo sapiens GN=RIPK3 PE=1 SV=2
Q9Y572	Receptor-interacting serine/threonine-protein kinase 3 OS=Homo sapiens GN=RIPK3 PE=1 SV=2
Q9Y572	Receptor-interacting serine/threonine-protein kinase 3 OS=Homo sapiens GN=RIPK3 PE=1 SV=2
Q9Y572	Receptor-interacting serine/threonine-protein kinase 3 OS=Homo sapiens GN=RIPK3 PE=1 SV=2
Q9Y572	Receptor-interacting serine/threonine-protein kinase 3 OS=Homo sapiens GN=RIPK3 PE=1 SV=2
Q9Y572	Receptor-interacting serine/threonine-protein kinase 3 OS=Homo sapiens GN=RIPK3 PE=1 SV=2
Q9Y572	Receptor-interacting serine/threonine-protein kinase 3 OS=Homo sapiens GN=RIPK3 PE=1 SV=2
Q9Y572	Receptor-interacting serine/threonine-protein kinase 3 OS=Homo sapiens GN=RIPK3 PE=1 SV=2
Q9Y572	Receptor-interacting serine/threonine-protein kinase 3 OS=Homo sapiens GN=RIPK3 PE=1 SV=2
Q9Y572	Receptor-interacting serine/threonine-protein kinase 3 OS=Homo sapiens GN=RIPK3 PE=1 SV=2
Q9Y572	Receptor-interacting serine/threonine-protein kinase 3 OS=Homo sapiens GN=RIPK3 PE=1 SV=2
Q9Y572	Receptor-interacting serine/threonine-protein kinase 3 OS=Homo sapiens GN=RIPK3 PE=1 SV=2
Q9Y572	Receptor-interacting serine/threonine-protein kinase 3 OS=Homo sapiens GN=RIPK3 PE=1 SV=2
Q9Y572	Receptor-interacting serine/threonine-protein kinase 3 OS=Homo sapiens GN=RIPK3 PE=1 SV=2
Q9Y572	Receptor-interacting serine/threonine-protein kinase 3 OS=Homo sapiens GN=RIPK3 PE=1 SV=2
Q9Y572	Receptor-interacting serine/threonine-protein kinase 3 OS=Homo sapiens GN=RIPK3 PE=1 SV=2
Q9Y572	Receptor-interacting serine/threonine-protein kinase 3 OS=Homo sapiens GN=RIPK3 PE=1 SV=2
Q9Y572	Receptor-interacting serine/threonine-protein kinase 3 OS=Homo sapiens GN=RIPK3 PE=1 SV=2
Q9Y572	Receptor-interacting serine/threonine-protein kinase 3 OS=Homo sapiens GN=RIPK3 PE=1 SV=2
Q9Y572	Receptor-interacting serine/threonine-protein kinase 3 OS=Homo sapiens GN=RIPK3 PE=1 SV=2
Q9Y572	Receptor-interacting serine/threonine-protein kinase 3 OS=Homo sapiens GN=RIPK3 PE=1 SV=2

Uniprot accession	description
Q9Y572	Receptor-interacting serine/threonine-protein kinase 3 OS=Homo sapiens GN=RIPK3 PE=1 SV=2
Q9Y572	Receptor-interacting serine/threonine-protein kinase 3 OS=Homo sapiens GN=RIPK3 PE=1 SV=2
Q9Y572	Receptor-interacting serine/threonine-protein kinase 3 OS=Homo sapiens GN=RIPK3 PE=1 SV=2
Q9Y572	Receptor-interacting serine/threonine-protein kinase 3 OS=Homo sapiens GN=RIPK3 PE=1 SV=2
Q9Y572	Receptor-interacting serine/threonine-protein kinase 3 OS=Homo sapiens GN=RIPK3 PE=1 SV=2
Q9Y572	Receptor-interacting serine/threonine-protein kinase 3 OS=Homo sapiens GN=RIPK3 PE=1 SV=2
Q9Y572	Receptor-interacting serine/threonine-protein kinase 3 OS=Homo sapiens GN=RIPK3 PE=1 SV=2
Q9Y572	Receptor-interacting serine/threonine-protein kinase 3 OS=Homo sapiens GN=RIPK3 PE=1 SV=2
Q9Y572	Receptor-interacting serine/threonine-protein kinase 3 OS=Homo sapiens GN=RIPK3 PE=1 SV=2
Q9Y572	Receptor-interacting serine/threonine-protein kinase 3 OS=Homo sapiens GN=RIPK3 PE=1 SV=2
Q9Y572	Receptor-interacting serine/threonine-protein kinase 3 OS=Homo sapiens GN=RIPK3 PE=1 SV=2
Q9Y572	Receptor-interacting serine/threonine-protein kinase 3 OS=Homo sapiens GN=RIPK3 PE=1 SV=2
Q9Y572	Receptor-interacting serine/threonine-protein kinase 3 OS=Homo sapiens GN=RIPK3 PE=1 SV=2
Q9Y572	Receptor-interacting serine/threonine-protein kinase 3 OS=Homo sapiens GN=RIPK3 PE=1 SV=2
Q9Y572	Receptor-interacting serine/threonine-protein kinase 3 OS=Homo sapiens GN=RIPK3 PE=1 SV=2
Q9Y572	Receptor-interacting serine/threonine-protein kinase 3 OS=Homo sapiens GN=RIPK3 PE=1 SV=2
Q9Y572	Receptor-interacting serine/threonine-protein kinase 3 OS=Homo sapiens GN=RIPK3 PE=1 SV=2
Q9Y572	Receptor-interacting serine/threonine-protein kinase 3 OS=Homo sapiens GN=RIPK3 PE=1 SV=2
Q9Y572	Receptor-interacting serine/threonine-protein kinase 3 OS=Homo sapiens GN=RIPK3 PE=1 SV=2
Q9Y572	Receptor-interacting serine/threonine-protein kinase 3 OS=Homo sapiens GN=RIPK3 PE=1 SV=2
Q9Y572	Receptor-interacting serine/threonine-protein kinase 3 OS=Homo sapiens GN=RIPK3 PE=1 SV=2
Q9Y572	Receptor-interacting serine/threonine-protein kinase 3 OS=Homo sapiens GN=RIPK3 PE=1 SV=2
Q9Y572	Receptor-interacting serine/threonine-protein kinase 3 OS=Homo sapiens GN=RIPK3 PE=1 SV=2
Q9Y572	Receptor-interacting serine/threonine-protein kinase 3 OS=Homo sapiens GN=RIPK3 PE=1 SV=2
Q9Y572	Receptor-interacting serine/threonine-protein kinase 3 OS=Homo sapiens GN=RIPK3 PE=1 SV=2
Q9Y572	Receptor-interacting serine/threonine-protein kinase 3 OS=Homo sapiens GN=RIPK3 PE=1 SV=2
Q9Y572	Receptor-interacting serine/threonine-protein kinase 3 OS=Homo sapiens GN=RIPK3 PE=1 SV=2
Q9Y572	Receptor-interacting serine/threonine-protein kinase 3 OS=Homo sapiens GN=RIPK3 PE=1 SV=2
Q9Y572	Receptor-interacting serine/threonine-protein kinase 3 OS=Homo sapiens GN=RIPK3 PE=1 SV=2
Q9Y572	Receptor-interacting serine/threonine-protein kinase 3 OS=Homo sapiens GN=RIPK3 PE=1 SV=2
Q9Y572	Receptor-interacting serine/threonine-protein kinase 3 OS=Homo sapiens GN=RIPK3 PE=1 SV=2

Uniprot accession	description
Q9Y572	Receptor-interacting serine/threonine-protein kinase 3 OS=Homo sapiens GN=RIPK3 PE=1 SV=2
Q9Y572	Receptor-interacting serine/threonine-protein kinase 3 OS=Homo sapiens GN=RIPK3 PE=1 SV=2
Q9Y572	Receptor-interacting serine/threonine-protein kinase 3 OS=Homo sapiens GN=RIPK3 PE=1 SV=2
Q9Y572	Receptor-interacting serine/threonine-protein kinase 3 OS=Homo sapiens GN=RIPK3 PE=1 SV=2
Q9Y572	Receptor-interacting serine/threonine-protein kinase 3 OS=Homo sapiens GN=RIPK3 PE=1 SV=2
Q9Y572	Receptor-interacting serine/threonine-protein kinase 3 OS=Homo sapiens GN=RIPK3 PE=1 SV=2
Q9Y572	Receptor-interacting serine/threonine-protein kinase 3 OS=Homo sapiens GN=RIPK3 PE=1 SV=2
Q9Y572	Receptor-interacting serine/threonine-protein kinase 3 OS=Homo sapiens GN=RIPK3 PE=1 SV=2
Q9Y572	Receptor-interacting serine/threonine-protein kinase 3 OS=Homo sapiens GN=RIPK3 PE=1 SV=2
Q9Y572	Receptor-interacting serine/threonine-protein kinase 3 OS=Homo sapiens GN=RIPK3 PE=1 SV=2
Q9Y572	Receptor-interacting serine/threonine-protein kinase 3 OS=Homo sapiens GN=RIPK3 PE=1 SV=2
Q9Y572	Receptor-interacting serine/threonine-protein kinase 3 OS=Homo sapiens GN=RIPK3 PE=1 SV=2
Q9Y572	Receptor-interacting serine/threonine-protein kinase 3 OS=Homo sapiens GN=RIPK3 PE=1 SV=2
Q9Y572	Receptor-interacting serine/threonine-protein kinase 3 OS=Homo sapiens GN=RIPK3 PE=1 SV=2

Uniprot accession	Experiment	Peptide Sequence	Modifications
P08238	3h Exp. 1	ADLINNLGTIAK	
P08238	3h Exp. 1	EQVANSAFVER	
P08238	3h Exp. 2	EQVANSAFVER	
P08238	3h Exp. 2	YIDQEELNK	
P19474	0h Exp. 1	ALCWVCAQSR	Carbamidomethyl (C6); Carbamidomethyl (C3)
P19474	0h Exp. 1	LQVALGELR	
P19474	0h Exp. 1	TCAVHITLDPDTANPWLILSEDRR	Carbamidomethyl (C2)
P19474	0h Exp. 2	CHSSALELLQEVIIVLER	Carbamidomethyl (C1)
P19474	0h Exp. 2	DLDITSPELR	
P19474	3h Exp. 1	ALCWVCAQSR	Carbamidomethyl (C6); Carbamidomethyl (C3)
P19474	3h Exp. 1	DHAMVPLEEAAQEYQEK	
P19474	3h Exp. 1	EAWDLGVCR	Carbamidomethyl (C8)
P19474	3h Exp. 1	LAQQSQALQELISELDRR	
P19474	3h Exp. 1	NTAPLTLCPLNIGSQGSTDY	Carbamidomethyl (C8)
P19474	3h Exp. 1	QLQELEKDER	
P19474	3h Exp. 1	SESWNLKDLDITSPELR	
P19474	3h Exp. 1	TCAVHITLDPDTANPWLILSEDRR	Carbamidomethyl (C2)
P19474	3h Exp. 2	ALCWVCAQSR	Carbamidomethyl (C6); Carbamidomethyl (C3)
P19474	3h Exp. 2	CHSSALELLQEVIIVLER	Carbamidomethyl (C1)
P19474	3h Exp. 2	DLDITSPELR	
P19474	3h Exp. 2	EAWDLGVCR	Carbamidomethyl (C8)
P19474	3h Exp. 2	HYWEVDVTGK	
P19474	3h Exp. 2	IHAEFVQQK	
P19474	3h Exp. 2	LAQQSQALQELISELDR	
P19474	3h Exp. 2	LGDTQQSIPGNEER	
P19474	3h Exp. 2	LHLFCEK	Carbamidomethyl (C5)
P19474	3h Exp. 2	LQVALGELR	
P19474	3h Exp. 2	NFLVEEEQR	
P19474	3h Exp. 2	NTAPLTLCPLNIGSQGSTDY	Carbamidomethyl (C8)
P19474	3h Exp. 2	QLQELEKDER	
P19474	3h Exp. 2	SGFWTIWLWNK	

Uniprot accession	Experiment	Peptide Sequence	Modifications
P19474	3h Exp. 2	TCAVHITLDPDTANPWLILSEDR	Carbamidomethyl (C2)
Q12933	3h Exp. 2	HLEHECPER	Carbamidomethyl (C6)
Q12933	3h Exp. 2	IPAIFSPAFYTSR	
Q12933	3h Exp. 2	RPFQAQCGHR	Carbamidomethyl (C7)
Q13158	3h Exp. 1	NTEKENATVAHLVGALR	
Q13158	3h Exp. 2	ENATVAHLVGALR	
Q13158	3h Exp. 2	IDSIEDRYPR	
Q13158	3h Exp. 2	NTEKENATVAHLVGALR	
Q13158	3h Exp. 2	RVDDFEAGAAAGAAPGEEDLCAAFNVICDNVGK	Carbamidomethyl (C28); Carbamidomethyl (C21)
Q13546	3h Exp. 1	FRPFYLSQLEESVEEDVK	
Q13546	3h Exp. 1	YQAIFDNTTSLTDK	
Q13546	3h Exp. 2	DLKPENILVDNDFHIK	
Q13546	3h Exp. 2	EYSNENAVVKR	
Q13546	3h Exp. 2	FRPFYLSQLEESVEEDVK	
Q13546	3h Exp. 2	IADLGLASFK	
Q13546	3h Exp. 2	KLGFTQSQIDEIDHDYER	
Q13546	3h Exp. 2	LAQALHQCSR	Carbamidomethyl (C8)
Q13546	3h Exp. 2	LGFTQSQIDEIDHDYER	
Q13546	3h Exp. 2	LLGVIIEEGK	
Q13546	3h Exp. 2	LNNEEHNELR	
Q13546	3h Exp. 2	SGNRPDVDDITEYCPR	Carbamidomethyl (C14)
Q13546	3h Exp. 2	YQAIFDNTTSLTDK	
Q14790	3h Exp. 1	FLSLDYIPQR	
Q14790	3h Exp. 1	MLEESNLSFLK	
Q14790	3h Exp. 1	NLYDIGEQLDSEDLASLK	
Q14790	3h Exp. 1	VMLYQISEEVSR	
Q14790	3h Exp. 2	FLLQEEISK	
Q14790	3h Exp. 2	FLSLDYIPQR	
Q14790	3h Exp. 2	INRLDLLITYLNTR	
Q14790	3h Exp. 2	LDLLITYLNTR	
Q14790	3h Exp. 2	NLYDIGEQLDSEDLASLK	

Uniprot accession	Experiment	Peptide Sequence	Modifications
Q14790	3h Exp. 2	VFFIQACQGDNYQK	Carbamidomethyl (C7)
Q15628	3h Exp. 2	CLSCILAQQPDR	Carbamidomethyl (C4); Carbamidomethyl (C1)
Q15628	3h Exp. 2	EGLYEQAFQLLR	
Q8NB16	0h Exp. 1	MPVSPISQGASWAQEDQQDADEDRR	
Q8NB16	3h Exp. 1	AALEEANGEIEK	
Q8NB16	3h Exp. 1	ELSLLLQVEQR	
Q8NB16	3h Exp. 1	MPVSPISQGASWAQEDQQDADEDRR	
Q8NB16	3h Exp. 1	QQEPLGEDCPSELR	Carbamidomethyl (C9)
Q8NB16	3h Exp. 1	QQEPLGEDCPSELREIIDECR	Carbamidomethyl (C20); Carbamidomethyl (C9)
Q8NB16	3h Exp. 1	SSNFLVTQGYQVK	
Q8NB16	3h Exp. 1	STAYLSPQELEDVFYQYDVK	
Q8NB16	3h Exp. 2	AALEEANGEIEK	
Q8NB16	3h Exp. 2	ELSLLLQVEQR	
Q8NB16	3h Exp. 2	LAGFELR	
Q8NB16	3h Exp. 2	LHHSEAPELHGK	
Q8NB16	3h Exp. 2	MPVSPISQGASWAQEDQQDADEDRR	
Q8NB16	3h Exp. 2	QQEPLGEDCPSELR	Carbamidomethyl (C9)
Q8NB16	3h Exp. 2	QQEPLGEDCPSELREIIDECR	Carbamidomethyl (C20); Carbamidomethyl (C9)
Q8NB16	3h Exp. 2	SSNFLVTQGYQVK	
Q8NB16	3h Exp. 2	STAYLSPQELEDVFYQYDVK	
Q96HS1	3h Exp. 1	AIETTDIISR	
Q96HS1	3h Exp. 1	EQAELTGLR	
Q96HS1	3h Exp. 1	QALQLAACGLAGGSAAVLFSAVAVGKPR	Carbamidomethyl (C8)
Q96HS1	3h Exp. 2	HSQYHVDGSLEK	
Q96HS1	3h Exp. 2	HSQYHVDGSLEKDR	
Q96HS1	3h Exp. 2	NVESGEEELASK	
Q96HS1	3h Exp. 2	QEEDSYEIFICHANVIR	Carbamidomethyl (C11)
Q9Y572	0h Exp. 1	AMASLDNEFVLR	
Q9Y572	0h Exp. 1	AQEEQVPQAWTAGTSSDSMAQPPQTPETSTFR	
Q9Y572	0h Exp. 1	DLKPSNVLLDPELHVK	
Q9Y572	0h Exp. 1	DRPSFQECLPK	Carbamidomethyl (C8)

Uniprot accession	Experiment	Peptide Sequence	Modifications
Q9Y572	0h Exp. 1	ELMQLCWSSEPK	Carbamidomethyl (C6)
Q9Y572	0h Exp. 1	EVELPTEPSLVYEAVCNR	Carbamidomethyl (C16)
Q9Y572	0h Exp. 1	EVVLGMFYLHDQNPVLLHR	
Q9Y572	0h Exp. 1	FMENGSLSGLLQSQCPRPWPLLCR	Carbamidomethyl (C23); Carbamidomethyl (C15); Oxidation (M2)
Q9Y572	0h Exp. 1	FMENGSLSGLLQSQCPRPWPLLCR	Carbamidomethyl (C23); Carbamidomethyl (C15)
Q9Y572	0h Exp. 1	FSIPESGQGGTEMDGFR	
Q9Y572	0h Exp. 1	GGFGTVFR	
Q9Y572	0h Exp. 1	KWGYDVAVK	
Q9Y572	0h Exp. 1	LADFGLSTFQGGSQSGTGSGEPGGTLGYLAPELFVNVNRK	
Q9Y572	0h Exp. 1	LLKEVVLGMFYLHDQNPVLLHR	
Q9Y572	0h Exp. 1	LNLEEPPSSVPK	
Q9Y572	0h Exp. 1	LWPSGAPAPLVSIEELENQELVGK	
Q9Y572	0h Exp. 1	NDVMVSEWLNK	
Q9Y572	0h Exp. 1	NDVMVSEWLNKLNLEEPPSSVPK	
Q9Y572	0h Exp. 1	NQMPSPTSTGTPSPGPR	Oxidation (M3)
Q9Y572	0h Exp. 1	NQMPSPTSTGTPSPGPR	
Q9Y572	0h Exp. 1	QGMNWSCR	Carbamidomethyl (C7)
Q9Y572	0h Exp. 1	QNRPSLAELPQAGPETPGLEGLK	
Q9Y572	0h Exp. 1	QNRPSLAELPQAGPETPGLEGLKELMQLCWSSEPK	Carbamidomethyl (C29)
Q9Y572	0h Exp. 1	TDEVFQMVENNMNAAVSTVK	
Q9Y572	0h Exp. 1	VNWDQDPKPALVTK	
Q9Y572	0h Exp. 1	WGYDVAVK	
Q9Y572	0h Exp. 2	AMASLDNEFVLR	
Q9Y572	0h Exp. 2	DFLSQLR	
Q9Y572	0h Exp. 2	DLKPSNVLLDPELHVK	
Q9Y572	0h Exp. 2	DRPSFQECLPK	Carbamidomethyl (C8)
Q9Y572	0h Exp. 2	EVELPTEPSLVYEAVCNR	Carbamidomethyl (C16)
Q9Y572	0h Exp. 2	FSIPESGQGGTEMDGFR	
Q9Y572	0h Exp. 2	LNLEEPPSSVPK	
Q9Y572	0h Exp. 2	LWPSGAPAPLVSIEELENQELVGK	
Q9Y572	0h Exp. 2	NDVMVSEWLNK	

Uniprot accession	Experiment	Peptide Sequence	Modifications
Q9Y572	0h Exp. 2	NQMPSPTSTGTPSPGPR	
Q9Y572	0h Exp. 2	QNRPSLAELPQAGPETPGLEGLK	
Q9Y572	0h Exp. 2	TDEVFQMVENNMNAAVSTVKDFLSQLR	
Q9Y572	0h Exp. 2	VNWDQDPKPALVTK	
Q9Y572	3h Exp. 1	AMASLDNEFVLR	
Q9Y572	3h Exp. 1	AMASLDNEFVLR	Oxidation (M2)
Q9Y572	3h Exp. 1	AQEEQVPQAWTAGTSSDSMAQPPQTPETSTFR	
Q9Y572	3h Exp. 1	DFLSQLR	
Q9Y572	3h Exp. 1	DLKPSNVLLDPELHVK	
Q9Y572	3h Exp. 1	DRPSFQECLPK	Carbamidomethyl (C8)
Q9Y572	3h Exp. 1	ELMQLCWSSEPK	Carbamidomethyl (C6)
Q9Y572	3h Exp. 1	EVELPTEPSLVYEAVCNR	Carbamidomethyl (C16)
Q9Y572	3h Exp. 1	EVVLGMFYLHDQNPVLLHR	
Q9Y572	3h Exp. 1	FMENGSLSGLLQSQCPRPWPLLCR	Carbamidomethyl (C23); Carbamidomethyl (C15); Oxidation (M2)
Q9Y572	3h Exp. 1	FMENGSLSGLLQSQCPRPWPLLCR	Carbamidomethyl (C23); Carbamidomethyl (C15)
Q9Y572	3h Exp. 1	FSIPESGQGGTEMDGFR	
Q9Y572	3h Exp. 1	GGFGTVFR	
Q9Y572	3h Exp. 1	KWGYDVAVK	
Q9Y572	3h Exp. 1	LEGVIEKVNWDQDPKPALVTK	
Q9Y572	3h Exp. 1	LLKEVVLGMFYLHDQNPVLLHR	
Q9Y572	3h Exp. 1	LNLEEPPSSVPK	
Q9Y572	3h Exp. 1	LWPSGAPAPLVSIEELENQELVGK	
Q9Y572	3h Exp. 1	NDVMVSEWLNK	
Q9Y572	3h Exp. 1	NDVMVSEWLNKLNLEEPPSSVPK	
Q9Y572	3h Exp. 1	NQMPSPTSTGTPSPGPR	Oxidation (M3)
Q9Y572	3h Exp. 1	NQMPSPTSTGTPSPGPR	
Q9Y572	3h Exp. 1	QGMNWSCR	Carbamidomethyl (C7)
Q9Y572	3h Exp. 1	QNRPSLAELPQAGPETPGLEGLK	
Q9Y572	3h Exp. 1	TDEVFQMVENNMNAAVSTVK	
Q9Y572	3h Exp. 1	VNWDQDPKPALVTK	
Q9Y572	3h Exp. 1	WGYDVAVK	

Uniprot accession	Experiment	Peptide Sequence	Modifications
Q9Y572	3h Exp. 2	ASTASDVYSFGILMWAVLAGR	
Q9Y572	3h Exp. 2	DLKPSNVLLDPELHVK	
Q9Y572	3h Exp. 2	DRPSFQECLPK	Carbamidomethyl (C8)
Q9Y572	3h Exp. 2	EVELPTEPSLVYEAVCNR	Carbamidomethyl (C16)
Q9Y572	3h Exp. 2	FMENGSLSGLLQSQCPRPWPLLCR	Carbamidomethyl (C23); Carbamidomethyl (C15)
Q9Y572	3h Exp. 2	KCPSLTK	Carbamidomethyl (C2)
Q9Y572	3h Exp. 2	KWGYDVAVK	
Q9Y572	3h Exp. 2	LNLEEPPSSVPK	
Q9Y572	3h Exp. 2	LWPSGAPAPLVSIEELENQELVGK	
Q9Y572	3h Exp. 2	QNRPSLAELPQAGPETPGLEGLK	
Q9Y572	3h Exp. 2	VNWDQDPKPALVTK	
Q9Y572	3h Exp. 2	WGYDVAVK	

Uniprot accession	Ion score	Experimental m/z	Charge	Calculated mass	Delta m/z	Miscleavages	Fragment matches count
P08238	23,56	621,8572	2	1241,6979	0,001003	0	13
P08238	39,95	625,3137	2	1248,6098	0,00146	0	6
P08238	43,9	625,3112	2	1248,6098	-0,00098	0	7
P08238	58,11	576,2813	2	1150,5506	-0,001258	0	7
P19474	30,41	625,7936	2	1249,5696	0,001506	0	6
P19474	22,68	499,8045	2	997,592	0,001249	0	6
P19474	19,02	699,1059	4	2792,3916	0,000735	1	10
P19474	72,11	1055,0734	2	2108,1299	0,001112	0	23
P19474	32,92	579,8021	2	1157,5928	-0,001618	0	9
P19474	32,4	625,7941	2	1249,5696	0,002056	0	9
P19474	32,49	994,4607	2	1986,8993	0,003784	0	16
P19474	33,81	553,2593	2	1104,5022	0,00095	0	6
P19474	29,05	700,0485	3	2097,1178	0,001923	1	11
P19474	42,71	1061,5135	2	2121,0048	0,003855	0	18
P19474	19,74	644,3315	2	1286,6466	0,000961	1	7
P19474	35,67	1002,0098	2	2002,0007	0,002203	1	11
P19474	30,31	699,1057	4	2792,3916	0,000545	1	11
P19474	35,1	625,7913	2	1249,5696	-0,000753	0	11
P19474	122,86	1055,0704	2	2108,1299	-0,001818	0	24
P19474	27,84	579,8019	2	1157,5928	-0,001738	0	8
P19474	53,27	553,2567	2	1104,5022	-0,00174	0	7
P19474	21,48	617,2972	2	1232,5826	-0,001334	0	7
P19474	42,2	550,2974	2	1098,5822	-0,000948	0	5
P19474	62,51	971,5145	2	1941,0167	-0,001145	0	10
P19474	45,56	772,3699	2	1542,7274	-0,001047	0	14
P19474	20,71	473,7428	2	945,4742	-0,001582	0	6
P19474	64,08	499,8017	2	997,592	-0,001611	0	8
P19474	30,19	582,2864	2	1162,5618	-0,001751	0	10
P19474	23,05	1061,5099	2	2121,0048	0,000196	0	11
P19474	49,24	644,3295	2	1286,6466	-0,001109	1	7
P19474	43,18	719,3703	2	1436,7241	0,000971	0	7

Uniprot accession	Ion score	Experimental m/z	Charge	Calculated mass	Delta m/z	Miscleavages	Fragment matches count
P19474	37,93	879,7694	3	2636,2905	-0,00135	0	14
Q12933	30,67	402,8483	3	1205,5247	-0,000584	0	7
Q12933	22,14	735,3914	2	1468,7714	-0,001576	0	5
Q12933	58,21	419,5404	3	1255,5993	0,000038	0	13
Q13158	36,34	608,3315	3	1821,9697	0,001037	1	16
Q13158	62,26	675,8758	2	1349,7415	-0,002252	0	8
Q13158	38,37	632,3186	2	1262,6255	-0,00142	1	10
Q13158	66,91	608,3293	3	1821,9697	-0,001153	1	13
Q13158	26,65	1137,1847	3	3408,5351	-0,000941	1	18
Q13546	53,25	739,0381	3	2214,0844	0,002667	0	9
Q13546	70,72	808,8962	2	1615,773	0,002482	0	12
Q13546	44,26	637,3373	3	1908,9945	-0,001438	0	10
Q13546	34,52	654,8307	2	1307,6469	0,000003	1	9
Q13546	24,29	739,034	3	2214,0844	-0,001423	0	15
Q13546	37,28	517,7957	2	1033,5808	-0,001946	0	7
Q13546	53,01	732,017	3	2193,0338	-0,001577	1	21
Q13546	64,6	592,3035	2	1182,5928	-0,000125	0	7
Q13546	51,38	689,318	3	2064,9389	-0,002179	0	12
Q13546	54,58	535,8247	2	1069,6383	-0,001771	0	14
Q13546	52,76	634,3049	2	1266,5952	0,000039	0	16
Q13546	49,15	947,4224	2	1892,8323	-0,001062	0	15
Q13546	95,93	808,8915	2	1615,773	-0,002278	0	9
Q14790	31,11	626,3411	2	1250,6659	0,000829	0	6
Q14790	29,92	655,8374	2	1309,6588	0,000748	0	5
Q14790	107,17	1012,0001	2	2021,9793	0,003197	0	13
Q14790	56,54	727,3737	2	1452,7282	0,002263	0	8
Q14790	57,83	553,8067	2	1105,6019	-0,001525	0	7
Q14790	64,39	626,3386	2	1250,6659	-0,001671	0	15
Q14790	38,31	859,5	2	1716,9886	-0,001584	1	14
Q14790	21,65	667,8858	2	1333,7605	-0,00174	0	8
Q14790	71,5	1011,9947	2	2021,9793	-0,002173	0	14

Uniprot accession	Ion score	Experimental m/z	Charge	Calculated mass	Delta m/z	Miscleavages	Fragment matches count
Q14790	51,9	859,402	2	1716,793	-0,001796	0	10
Q15628	35,5	730,8517	2	1459,6912	-0,001177	0	13
Q15628	36,19	733,8848	2	1465,7565	-0,000696	0	6
Q8NB16	38,69	939,4252	3	2815,2467	0,002376	1	10
Q8NB16	24,87	637,3187	2	1272,6197	0,001532	0	5
Q8NB16	25,61	664,3834	2	1326,7507	0,0008	0	6
Q8NB16	42,4	939,4254	3	2815,2467	0,002556	1	10
Q8NB16	58,66	829,381	2	1656,7413	0,003097	0	9
Q8NB16	24,01	858,3945	3	2572,1533	0,002749	1	6
Q8NB16	56,95	735,8848	2	1469,7514	0,001769	0	8
Q8NB16	34,12	1198,0753	2	2394,1267	0,00469	0	7
Q8NB16	71,97	637,3151	2	1272,6197	-0,002069	0	10
Q8NB16	22,87	664,3815	2	1326,7507	-0,00115	0	5
Q8NB16	22,05	403,2312	2	804,4494	-0,000791	0	5
Q8NB16	46,81	452,2332	3	1353,6789	-0,000374	0	10
Q8NB16	48,38	939,4208	3	2815,2467	-0,002014	1	15
Q8NB16	65,95	829,3762	2	1656,7413	-0,001782	0	10
Q8NB16	26,04	858,3903	3	2572,1533	-0,001461	1	11
Q8NB16	62,43	735,8817	2	1469,7514	-0,001351	0	15
Q8NB16	63,52	1198,0712	2	2394,1267	0,000539	0	9
Q96HS1	42	559,8088	2	1117,5979	0,002562	0	8
Q96HS1	26,35	508,7733	2	1015,5298	0,001144	0	7
Q96HS1	19,83	895,1667	3	2682,4639	0,004771	0	8
Q96HS1	44,47	700,3329	2	1398,6528	-0,000779	0	18
Q96HS1	54,04	557,6006	3	1669,7808	-0,000239	1	10
Q96HS1	43,22	646,3021	2	1290,5939	-0,002168	0	9
Q96HS1	49,37	708,3318	3	2121,9789	-0,001795	0	11
Q9Y572	39,73	683,3464	2	1364,6758	0,001188	0	8
Q9Y572	20,49	1155,1985	3	3462,5634	0,003414	0	19
Q9Y572	47,07	606,345	3	1816,0094	0,001217	0	11
Q9Y572	36,92	688,8369	2	1375,6554	0,001921	0	8

Uniprot accession	Ion score	Experimental m/z	Charge	Calculated mass	Delta m/z	Miscleavages	Fragment matches count
Q9Y572	25,94	754,3514	2	1506,6847	0,001833	0	5
Q9Y572	48,37	702,3483	3	2104,0146	0,002838	0	12
Q9Y572	33,22	760,7388	3	2279,1885	0,001997	0	15
Q9Y572	34,96	954,8024	3	2861,3775	0,002593	0	13
Q9Y572	28,5	949,47	3	2845,3826	0,001893	0	9
Q9Y572	46,29	907,9066	2	1813,7941	0,00228	0	11
Q9Y572	38,64	420,7227	2	839,429	0,000936	0	5
Q9Y572	34,21	533,2914	2	1064,5655	0,001427	1	4
Q9Y572	75,04	1343,6782	3	4027,9916	0,007075	1	18
Q9Y572	54,79	878,8276	3	2633,4516	0,003174	1	16
Q9Y572	65,18	655,3553	2	1308,6925	0,001762	0	8
Q9Y572	22,14	1288,696	2	2575,3533	0,012098	0	8
Q9Y572	37,44	667,8259	2	1333,6336	0,001843	0	11
Q9Y572	44,08	1313,1685	2	2624,3156	0,0034	1	7
Q9Y572	52,88	864,406	2	1726,7945	0,001502	0	8
Q9Y572	42,44	856,4096	2	1710,7995	0,002562	0	9
Q9Y572	34,59	519,7164	2	1037,4171	0,000597	0	7
Q9Y572	33,72	1201,6403	2	2401,2601	0,002934	0	8
Q9Y572	26,8	973,4934	4	3889,9342	0,002583	1	17
Q9Y572	86,67	1114,0256	2	2226,0297	0,003522	0	14
Q9Y572	53,71	805,933	2	1609,8464	0,002562	0	16
Q9Y572	46,74	469,2432	2	936,4705	0,000684	0	7
Q9Y572	31,49	683,3443	2	1364,6758	-0,000882	0	7
Q9Y572	21,54	439,7388	2	877,4658	-0,001325	0	3
Q9Y572	55,16	909,0095	2	1816,0094	-0,002531	0	12
Q9Y572	40,66	688,8333	2	1375,6554	-0,001739	0	8
Q9Y572	89,94	1053,0145	2	2104,0146	-0,00007	0	22
Q9Y572	50,83	907,9016	2	1813,7941	-0,00273	0	11
Q9Y572	71,85	655,3523	2	1308,6925	-0,001239	0	15
Q9Y572	49,1	1288,6838	2	2575,3533	-0,000103	0	20
Q9Y572	24,15	667,822	2	1333,6336	-0,002127	0	10

Uniprot accession	Ion score	Experimental m/z	Charge	Calculated mass	Delta m/z	Miscleavages	Fragment matches count
Q9Y572	75,71	856,4052	2	1710,7995	-0,001839	0	13
Q9Y572	41,12	801,425	3	2401,2601	-0,00232	0	18
Q9Y572	27,47	1029,5038	3	3085,4849	0,00155	1	23
Q9Y572	65,59	805,9291	2	1609,8464	-0,001399	0	9
Q9Y572	61,3	683,3464	2	1364,6758	0,001258	0	9
Q9Y572	21,53	691,3434	2	1380,6707	0,000738	0	9
Q9Y572	21,74	1155,1984	3	3462,5634	0,003284	0	20
Q9Y572	29,45	439,7408	2	877,4658	0,000624	0	4
Q9Y572	39,38	909,0142	2	1816,0094	0,002169	0	10
Q9Y572	24,02	459,5599	3	1375,6554	0,000795	0	11
Q9Y572	48,62	754,3516	2	1506,6847	0,002013	0	8
Q9Y572	86,47	1053,0181	2	2104,0146	0,00347	0	10
Q9Y572	42,7	760,7383	3	2279,1885	0,001507	0	8
Q9Y572	24,35	954,8025	3	2861,3775	0,002713	0	11
Q9Y572	34,04	949,4708	3	2845,3826	0,002623	0	9
Q9Y572	45,56	907,9066	2	1813,7941	0,00228	0	7
Q9Y572	40,38	420,7227	2	839,429	0,000906	0	5
Q9Y572	21,54	533,2909	2	1064,5655	0,000936	1	4
Q9Y572	25,09	793,7707	3	2378,2845	0,001899	1	13
Q9Y572	36,87	878,8271	3	2633,4516	0,002624	1	20
Q9Y572	58,05	655,3553	2	1308,6925	0,001762	0	15
Q9Y572	38,99	859,4648	3	2575,3533	0,006393	0	16
Q9Y572	57,59	667,8254	2	1333,6336	0,001353	0	13
Q9Y572	32,68	875,7826	3	2624,3156	0,003458	1	8
Q9Y572	32,54	864,4055	2	1726,7945	0,000952	0	6
Q9Y572	45,66	856,4081	2	1710,7995	0,001092	0	9
Q9Y572	35,95	519,7166	2	1037,4171	0,000777	0	7
Q9Y572	64,19	801,4296	3	2401,2601	0,00226	0	9
Q9Y572	48,62	1114,0259	2	2226,0297	0,003772	0	8
Q9Y572	54,78	805,934	2	1609,8464	0,003482	0	11
Q9Y572	48,69	469,2435	2	936,4705	0,000994	0	7

Uniprot accession	Ion score	Experimental m/z	Charge	Calculated mass	Delta m/z	Miscleavages	Fragment matches count
Q9Y572	70,6	1108,0654	2	2214,1143	0,000995	0	11
Q9Y572	53,91	606,3423	3	1816,0094	-0,001473	0	9
Q9Y572	38,38	688,8323	2	1375,6554	-0,002709	0	7
Q9Y572	79,48	1053,0148	2	2104,0146	0,00017	0	9
Q9Y572	30,01	949,4668	3	2845,3826	-0,001337	0	7
Q9Y572	23,09	417,231	2	832,4477	-0,000117	1	9
Q9Y572	20,66	533,2874	2	1064,5655	-0,002603	1	9
Q9Y572	69,9	655,3525	2	1308,6925	-0,001048	0	13
Q9Y572	44,76	1288,6869	2	2575,3533	0,002947	0	18
Q9Y572	59,21	801,4254	3	2401,2601	-0,0019	0	21
Q9Y572	61,84	805,9285	2	1609,8464	-0,002009	0	18
Q9Y572	20,2	469,2414	2	936,4705	-0,001086	0	5
